# Cadherin adhesion complexes direct cell aggregation in the epithelial transition of Wnt-induced nephron progenitor cells

**DOI:** 10.1242/dev.202303

**Published:** 2024-09-30

**Authors:** Balint Der, Helena Bugacov, Bohdana-Myroslava Briantseva, Andrew P. McMahon

**Affiliations:** ^1^ Department of Stem Cell Biology and Regenerative Medicine, Eli and Edythe Broad Center for Regenerative Medicine and Stem Cell Research, Keck School of Medicine, University of Southern California, Los Angeles 90033, USA; ^2^ Department of Urology, Faculty of Medicine, Semmelweis University, Budapest 1082, Hungary; ^3^ Institute of Translational Medicine, Faculty of Medicine, Semmelweis University, Budapest 1094, Hungary; ^4^ Icahn School of Medicine at Mount Sinai, New York, NY 10029, USA

**Keywords:** Kidney, Nephron progenitor, Renal vesicle, Wnt, β-Catenin, Induction, Cadherin cell adhesion

## Abstract

In the developing mammalian kidney, nephron formation is initiated by a subset of nephron progenitor cells (NPCs). Wnt input activates a β-catenin (*Ctnnb1*)-driven, transcriptional nephrogenic program and the mesenchymal to epithelial transition (MET) of NPCs. Using an *in vitro* mouse NPC culture model, we observed that activation of the Wnt pathway results in the aggregation of induced NPCs, which is an initiating step in the MET program. Genetic removal showed aggregation was dependent on β-catenin. Modulating extracellular Ca^2+^ levels showed cell-cell contacts were Ca^2+^ dependent, suggesting a role for cadherin (Cdh)-directed cell adhesion. Molecular analysis identified *Cdh2*, *Cdh4* and *Cdh11* in NPCs, and the β-catenin directed upregulation of *Cdh3* and *Cdh4* accompanying the MET of induced NPCs. Mutational analysis of β-catenin supported a role for a Lef/Tcf-β-catenin-mediated transcriptional response in the cell aggregation process. Genetic removal of all four cadherins, and independent removal of α-catenin or of β-catenin-α-catenin interactions, abolished aggregation, but not the inductive response to Wnt pathway activation. These findings, and data in an accompanying article highlight the role of β-catenin in linking transcriptional programs to the morphogenesis of NPCs in mammalian nephrogenesis.

## Teaser

**Summary:** Stabilization of β-catenin on Wnt-mediated induction of mammalian nephron progenitors triggers cadherin-dependent cell aggregation at the onset of a mesenchymal-to-epithelial transition establishing the nephron anlagen.

## INTRODUCTION

The developmental morphogenesis of complex tissues requires the coordinated action of distinct cell behaviors (Gumbiner, 1996). In the mammalian kidney, the induction of mesenchymal nephron progenitor cells (NPCs), which initiates a nephrogenic developmental program, is coupled to the establishment of an epithelial nephron precursor, the renal vesicle (RV; McMahon, 2016). The developmental routines of NPC induction and mesenchymal-to-epithelial transition (MET) continue for days (mouse) to weeks (human), in conjunction with the expansion of the starting pool of NPCs. Consequently, the formation of a species-appropriate complement of nephrons – around 14,000 nephrons in the mouse and one million in the human kidney (McMahon, 2016; Schnell et al., 2022; Short et al., 2014) – is dependent on an orchestrated set of cellular processes. Furthermore, NPC development is closely linked to parallel development of the adjacent ureteric progenitor cells (UPCs) of the collecting system (Shakya et al., 2005) and interstitial (stromal) progenitor cells (Wilson and Little, 2021).

Multiple evidence has highlighted the crucial role of canonical Wnt9b/β-catenin (*Ctnnb1*)-mediated signaling in the primary induction of NPCs (Guo et al., 2021; Park et al., 2007; Ramalingam et al., 2018). Transduction of UPC-derived Wnt9b signals by a subset of overlying NPCs results in the accumulation of β-catenin (*Ctnnb1*), which associates with Lef/Tcf DNA-binding complexes to transcriptionally activate Wnt targets (Carroll et al., 2005; Park et al., 2012). *In vivo* analysis and *in vitro* modeling of these events in primary NPC culture have identified Lef/Tcf/β-catenin-dependent regulators of the nephrogenic program and demonstrated the direct interaction of Lef/Tcf/β-catenin complexes with cis-regulatory modules that regulate target gene expression (Guo et al., 2021; Park et al., 2012). In parallel with transcriptional activation of the nephrogenic program, induced NPCs cluster and condense into pre-tubular aggregates (PTAs), which complete a MET that establishes epithelial renal vesicles, which are precursors to the functional nephrons of the mammalian kidney (Georgas et al., 2009).

The molecular mechanism governing morphological transition of mesenchymal NPCs into an epithelial nephron anlagen are unclear. β-Catenin plays a crucial role in cadherin-mediated cell-adhesion complexes (Halbleib and Nelson, 2006) and is consequently an attractive candidate for promoting the aggregation and epithelial transition of NPCs. However, constitutive elevation of β-catenin levels blocks epithelial formation *in vitro* (Park et al., 2012) and several lines of evidence suggest a non-canonical signaling role for Wnt4, a primary transcriptional target of Wnt9b/β-catenin induced NPCs in the MET (Saulnier et al., 2002; Stark et al., 1994; Tanigawa et al., 2011; Vainio et al., 1999). Furthermore, in the gastrulating vertebrate embryo (Kelly et al., 2004; Liu et al., 1999) and in metastasis of a variety of epithelial cancers (Dongre and Weinberg, 2019; Huang et al., 2022), canonical Wnt signaling is linked to an opposite cellular program: an epithelial-to-mesenchymal transition (EMT).

Various observational and genetic studies have explored the potential role of cadherin complexes in the morphogenesis of the nephron during the MET. Several cadherins have been reported within NPCs, renal vesicles and the later nephron anlagen to the S-shaped body stage, including Cdh1 (Cho et al., 1998), Cdh2 (Naiman et al., 2017), Cdh3 (Goto et al., 1998; Lefevre et al., 2017), Cdh4 (Dahl et al., 2002) and Cdh6 (K-cadherin, (Cho et al., 1998). Mutants lacking Cdh4 showed reduced nephron formation (Dahl et al., 2002). However, interpreting Cdh4 function was complicated by deficiencies within the adjacent ureteric epithelium, the source of nephron-inducing Wnt9b signal (Carroll et al., 2005). Furthermore, examination of kidneys eliminating or reducing additional cadherin members (*Cdh2^+/−^; Cdh3^−/−^; Cdh4^−/−^; Cdh6^−/−^*) failed to enhance the *Cdh4* mutant phenotype*.*

Here, we have used cell imaging, cell profiling and molecular-genetic approaches to characterize the morphological changes associated with Wnt-β-catenin mediated induction of NPCs *in vitro*. These studies, together with those in the accompanying article (Bugacov et al., 2024), demonstrate that distinct actions of β-catenin in transcriptional regulation and cell-cell adhesion coordinate gene regulatory and morphological cellular programs that direct early mammalian nephrogenesis.

## RESULTS

### Increasing Wnt/β-catenin activity in NPCs *in vitro* models NPC induction and morphogenesis *in vivo*

**Fig. 1.**
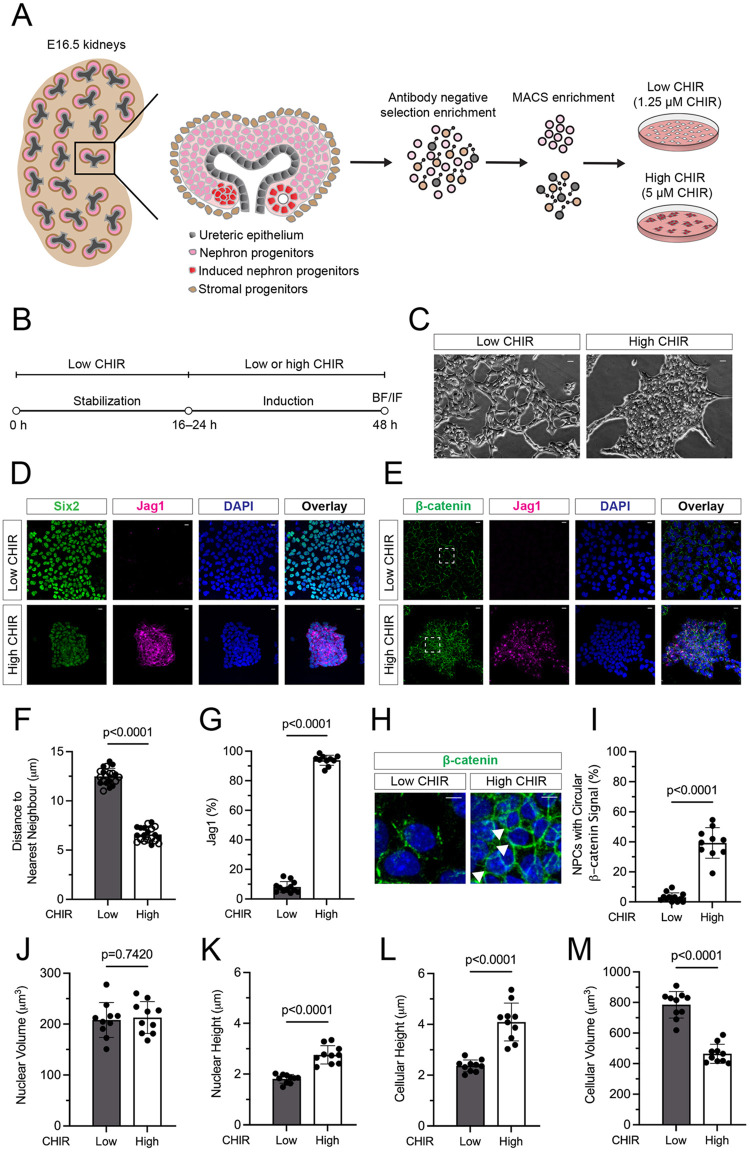
**Nephron progenitor cells undergo morphological and transcriptional changes *in vitro* as a result of increased Wnt stimulus.** (A) Schematic representation of nephron progenitor cell (NPC) isolation and culture system in nephron progenitor expansion media (NPEM) supplemented with low (1.25 μM) CHIR or high (5 μM) CHIR. (B) Schematic diagram of experimental protocol. BF, bright field; IF, immunofluorescence. (C) Phase-contrast images of dispersed NPCs in low CHIR conditions versus NPC aggregates in high CHIR conditions. Scale bars: 50 μm. (D) Immunofluorescence staining of isolated E16.5 NPCs characterizing Six2 (green), Jag1 (magenta) and nuclear DNA (DAPI, blue). Scale bars: 10 μm. (E) Immunofluorescence staining of isolated E16.5 NPCs characterizing β-catenin (green), Jag1 (magenta) and DAPI (blue). Outlined areas are shown at higher magnification in H. Scale bars: 10 μm. (F) Quantification of cell aggregation by plotting distance to nearest neighbor (unpaired *t*-test; two technical replicates plotted as differently filled circles). (G) Quantification of induction by the percentage of Jag1^+^ immunopositive NPCs (unpaired *t*-test). (H) Representative images of continuous membrane β-catenin (Ctnnb1) (green, arrowheads) in aggregated NPCs in high CHIR conditions (DAPI^+^ nuclei, blue). In low CHIR conditions, Ctnnb1 is discontinuous at the cell membrane. The areas shown at high magnification are from areas indicated in E. (I) Quantification of Ctnnb1 membrane distribution (unpaired *t*-test). (J-M) Quantification of cell morphology changes of E16.5 wild-type NPCs cultured in low CHIR and high CHIR conditions. Differences in nuclear volume (J), nuclear height (K), cellular height (L) and cellular volume (M) were all tested for significance using an unpaired *t*-test. Data are mean±s.d.

To examine the mechanisms of canonical Wnt pathway action on NPC programs, we used an *in vitro* model comprising highly purified naïve uninduced mouse NPCs cultured in a defined nephron progenitor expansion media (NPEM). NPEM replicates multiple signaling activities linked to the maintenance and expansion of NPCs in the mouse kidney (Brown et al., 2015). As shown in the accompanying article (Bugacov et al., 2024), NPC fate outcomes in this model are dependent on the concentration of CHIR99021 (hereafter CHIR), a small molecule antagonist of the serine-threonine kinase GSK3β (Fig. 1A,B). As, GSK3β-mediated phosphorylation of β-catenin results in β-catenin turnover by a destruction complex (Bennett et al., 2002), varying degrees of stabilization of β-catenin mirror dose-dependent, Wnt-receptor-mediated canonical Wnt signaling (Fig. 1B). Low CHIR (1.25 µM) maintained dispersed NPCs in a Six2^high^/Jag1^−^ state (Fig. 1C,D), whereas high CHIR (5 µM) induced the transcriptional activation of a nephrogenic program (for a more complete analysis, see Bugacov et al., 2024) and the aggregation of induced Six2^low^/Jag1^+^ NPCs into tightly adherent multicellular clusters (Fig. 1C,D). Aggregates showed enhanced cell membrane-associated accumulation of β-catenin in a continuous ring (Fig. 1E; arrows in Fig. 1H, quantification is Fig. 1I). Consequently, nearest cell neighbor distance decreased in high CHIR (Fig. 1F), in conjunction with induction of Jag1, which is an early and direct transcriptional response to canonical Wnt complexes (Fig. 1G; Guo et al., 2021). Although nuclear volume was unaltered in high CHIR-induced aggregates (Fig. 1J), nuclear height and cell height increased (Fig. 1K,L), and overall cell volume decreased (Fig. 1M). Thus, 24 h after the switch from low to high CHIR, NPCs morphologically transform from flattened mesenchyme to columnar multicellular aggregates, which is reflective of PTA formation *in vivo*.

### High-resolution timelapse imaging of cell contact stabilization on induction of NPCs

To investigate the dynamics of cell aggregation, NPCs labeled with a cell membrane targeted tdTomato fluorescent protein (Muzumdar et al., 2007) were cultured in the absence of CHIR, or in low or high CHIR conditions and followed by time-lapse confocal microscopy over 6 h. Increasing CHIR levels resulted in increased clustering of NPCs: clustering was tightest and cell-cell adhesions most stable in high-CHIR conditions (Movie 1, Fig. S1A). Consequently, NPCs cultured in high CHIR showed the shortest average distance to their nearest neighbors (Fig. S1B) and experienced fewer contacts per cell over the imaging time course (Fig. S1C). Thus, NPCs are more motile and maintain shorter cell-cell contacts in both the absence of CHIR or in low CHIR, whereas high-CHIR conditions stabilized cell-cell contacts (Fig. S1D). In all conditions, NPCs send out filopodia that result in cell-cell contacts. An increased membrane surface contact in high CHIR (Fig. S1E) correlated with an increased stability of filopodia interactions between cells in high CHIR, which was evident as early as 3 h after initiation of high CHIR culture (Fig. S1F-H).

### β-Catenin activity is required for cell-cell aggregation of induced NPCs

**Fig. 2.**
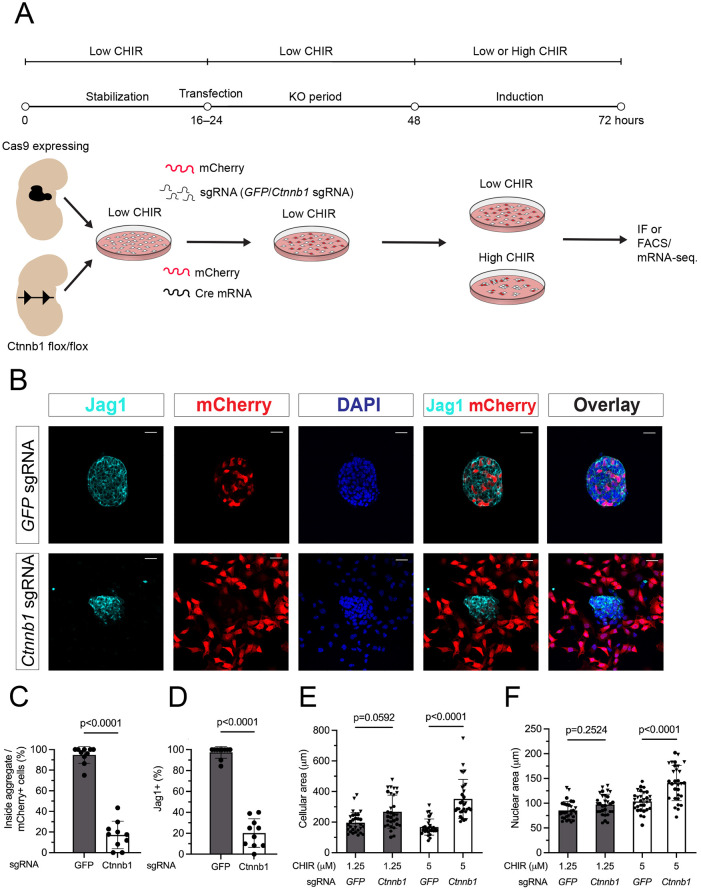
***In vitro* deletion of β-catenin results in nephron progenitor cell sorting and abolishes the CHIR-dependent induction program.** (A) Schematic of the experimental protocol in β-catenin KO experiments with a 24 h KO period and 24 h induction time for B-F. (B) Representative images of CTRL (GFP sgRNA) and β-catenin-KO (Ctnnb1 sgRNA) conditions showing loss of cell aggregation and Jag1 induction in mCherry^+^ nephron progenitor cells (NPCs) on loss of Ctnnb1 in high CHIR conditions. Scale bars: 25 μm. (C,D) Quantification of cell-sorting phenomenon by percentage of cells within aggregates (C) and induction by percentage of Jag1^+^ NPCs (D) as a result of knocking out β-catenin in NPCs. (Mann–Whitney test). (E,F) Quantification of changes in cellular morphology as a result of β-catenin KO in NPCs. Differences in cellular area were tested for significance using a ordinary one-way ANOVA; differences in nuclear area were tested for significance using a Kruskall-Wallis test. Data are mean±s.d.

To examine the requirement for β-catenin in cell-cell aggregation, RNA-lipofection was used to remove *Ctnnb1* function and β-catenin activity by either sgRNA/Cas9-directed gene knockout (KO) or CRE-directed critical exon excision, identifying genetically modified cells through co-activation of a mCherry reporter [Fig. 2A; for details see accompanying article by Bugacov et al. (2024)]. As expected, loss of β-catenin blocked high CHIR-mediated induction of Jag1, but also resulted in a failure in cell-cell aggregation (Fig. 2B,C-F). Exclusion of mutant cells could be observed 6-12 h after addition of high CHIR (Fig. S2A,B, live imaging in Movie 2). Addition of a bi-specific antibody (BSAB), which mediated direct activation of cell-surface Wnt-receptors (Janda et al., 2017), produced an indistinguishable outcome from high CHIR (Fig. S2B).

To obtain a better insight into the dynamics of β-catenin-KO NPCs, we focused on control gRNA and on β-catenin gRNA-transfected β-catenin-KO NPCs at the edges of colonies at 6-12 h into high CHIR induction (Movie 3, Fig. S2C). Whereas wild-type NPCs in control transfections maintained adherence to the larger cell aggregate, β-catenin-KO NPCs did not form longer term stable contacts and moved along the edges of cell aggregates, eventually moving away from cell-cell aggregates. In summary, β-catenin activity was required for stabilizing cell-cell associations that were essential for the generation of multicellular aggregates in conjunction with transcriptional activation of canonical Wnt signaling.

### Functional analysis of α-catenin supports independent actions of β-catenin in transcriptional regulation and aggregation of NPCs

In Ca^2+^ mediated, cadherin-directed cell-cell adhesion, β-catenin interactions with membrane-bound cadherins are relayed to the cytoskeleton through *α*-catenin (Aberle et al., 1994; Maiden and Hardin, 2011). Culturing NPCs in media without Ca^2+^, in either low- or high-CHIR conditions, led to a rapid loss of cell-cell contacts within 5 min (Fig. S2D,E), which could be reversed by the addition of Ca^2+^, although over a longer time course (30 min). The results support a role for Ca^2+^-dependent cadherin-catenin complex in aggregation of NPCs, although it should be noted that Ca^2+^ removal has also been shown to influence integrin functions (Kirchhofer et al., 1991).

**Fig. 3.**
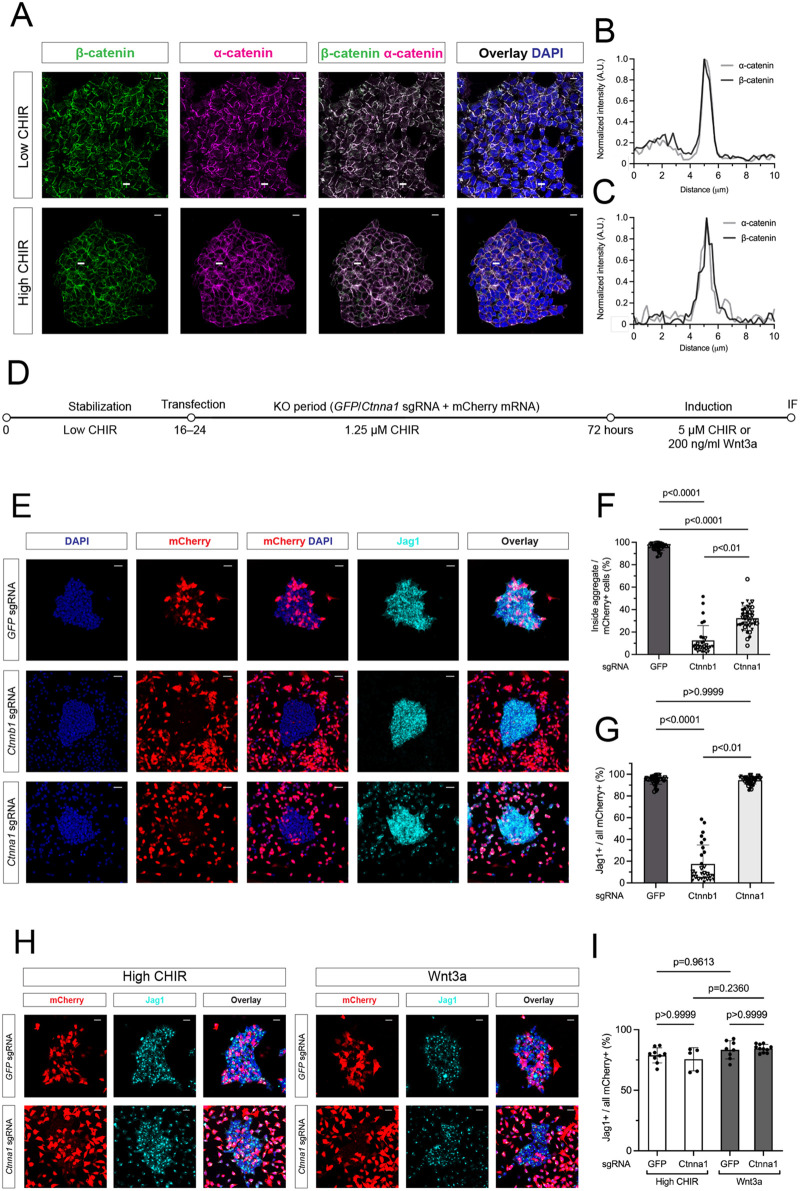
**Removal of α-catenin in nephron progenitor cells in high CHIR conditions phenocopies β-catenin KO-dependent cell sorting, but induction of Jag1 was not altered.** (A) Representative images of isolated E16.5 nephron progenitor cell (NPC) immunofluorescence co-staining of α- and β-catenin (α-catenin, magenta; β-catenin, green) in low and high CHIR conditions. Intensity values over the in-frame lines are plotted in B and C. Scale bars: 10 μm. (B,C) Normalized fluorescent intensity (normalization to baseline values) showing highly overlapping intensity values for α- and β-catenin. (D) Schematic of the experimental protocol in α-catenin KO experiments corresponding to E-I. (E) Representative images of CTRL (GFP sgRNA), β-and α-catenin-KO conditions showing cellular behavior (mCherry, DAPI) and the inductive response (Jag1) in 5 μM CHIR. Scale bars: 25 μm. (F,G) Quantification of cell-sorting (F) and the percentage of Jag1^+^ cells in aggregates (G) in high CHIR conditions analyzed using a Kruskal–Wallis test. Biological replicates are represented by different symbol shapes and technical replicates are represented by different colored symbols. (H) Representative images of CTRL (GFP sgRNA) and α-catenin-KO conditions showing altered cell aggregation but normal Jag1 (cyan) induction in α-catenin-KO transfected (mCherry) cells (DAPI, blue nuclear staining) treated with 200 ng/ml Wnt3a. Scale bars: 20 μm. (I) Quantification of inductive response in H as a percentage of transfected mCherry^+^/Jag1^+^ NPCs (Kruskall-Wallis test). Data are mean±s.d.

Next, we examined a potential role for α-catenin in the MET. *In vivo* in developing E16.5 kidney, β-catenin and α-catenin colocalize within Jag1^+^ cells in induced aggregating NPCs (Fig. S3A) identically to the *in vitro* culture system (Fig. 3A-C). To examine the function of α-catenin, we used α-catenin sgRNA and gene editing in Cas9^+^ NPCs to remove the function of the α-catenin-encoding gene *Ctnna1*, extending the KO period to 48 h for efficient removal of the α-catenin protein (∼95% removal; Fig. S3B-D). Strikingly, although α-catenin removal blocked cell aggregation in high CHIR, similar to the removal of β-catenin, α-catenin KO cells induced Jag1, which is indicative of an active canonical Wnt-directed transcriptional response (Fig. 3D-G). Thus, α-catenin separates distinct actions for β-catenin in the NPC inductive program. Replacing high CHIR with Wnt3a produced a similar inductive response (Fig. S3E) and cell distribution, indicating that the α-catenin-dependent cell aggregation was a bona fide outcome of canonical Wnt pathway activation (Fig. 3H,I).

### Multiple cadherins mediate NPC aggregation on canonical Wnt pathway activation

**Fig. 4.**
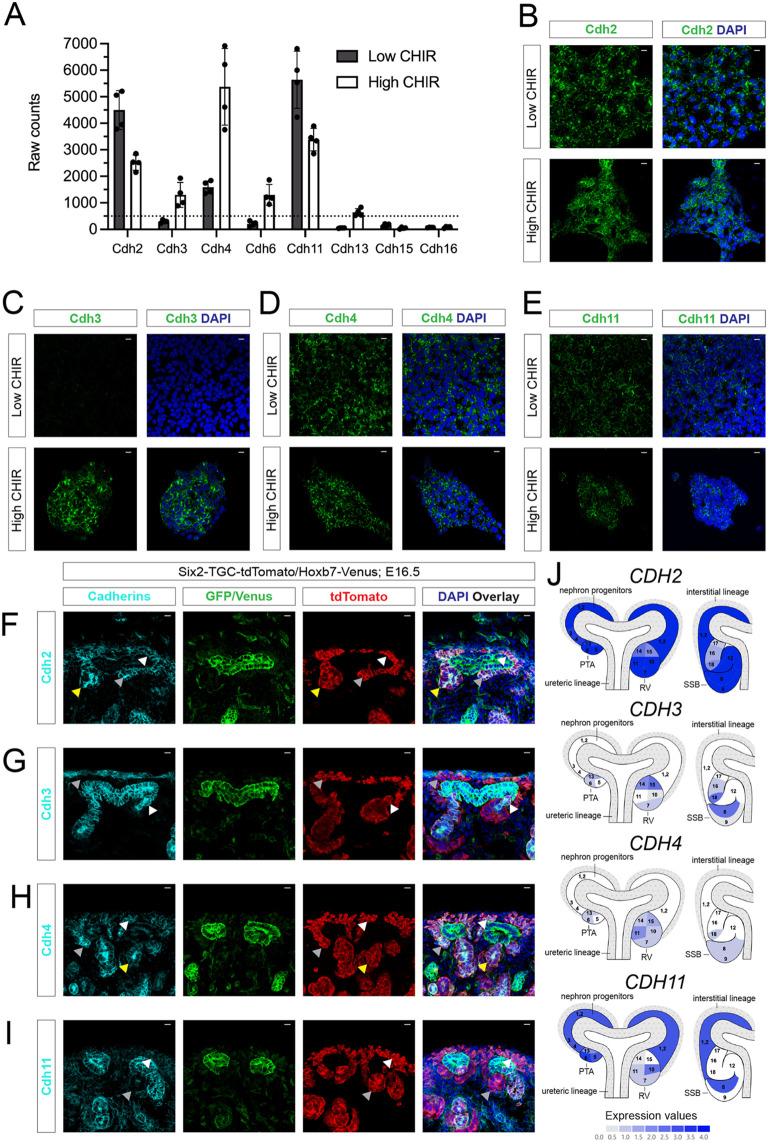
***In vitro* and *in vivo* analysis of cadherin mRNA and protein levels reveals that nephron progenitor cell culture system models the *in vivo* conditions.** (A) Raw counts of bulk RNA-seq for selected cadherin mRNAs in E16.5 nephron progenitor cells (NPCs) following 24 h of culture in low and high CHIR conditions. Data are mean±s.d. (B-E) immunofluorescence staining of isolated E16.5 NPCs showing Cdh2 (B) and Cdh4 (D) in the cell membrane in low and high CHIR conditions. High levels of Cdh3 (C) were restricted to the high CHIR condition; weak membrane Cdh11 labeling (E) was restricted to low and high CHIR conditions. Scale bars: 10 μm. (F-I) Representative images of immunofluorescence staining of E16.5 Six2-TGC-tdTomato/Hoxb7-Venus mouse kidneys highlighting indicated cadherins (cyan), tdTomato (nephron lineage, red) and GFP: nuclear GFP highlights Six2 in NPCs, while membrane GFP labels Venus reporter in the ureteric lineage. (F) Cdh2 is present in both uninduced and induced NPCs (white and gray arrowheads, respectively), and late RVs (yellow arrowheads). (G) Cdh3 is present in the distal segment of late RV, including the invading cells of the ureteric bud tip (white arrowheads), but not in the uninduced NPCs (gray arrowheads). (H) Cdh4 is present in uninduced NPCs (white arrowheads) and at higher levels in the PTA and SSB (gray and yellow arrowheads, respectively). (I) Cdh11 is present at low levels in uninduced NPCs (white arrowheads) and levels decrease in the RV (gray arrowheads). Scale bars: 10 μm. (J) Human Nephrogenesis Atlas views (https://sckidney.flatironinstitute.org/) of human CDH gene expression in early development of the human nephron lineage. PTA, pre-tubular aggregate; RV, renal vesicle; SSB, S-shaped body.

Together, the findings above suggest a role for cadherin-catenin complexes in the cell-cell aggregation of induced NPCs. Previous reports examining single and multiple allelic combinations of cadherin mutants (Cdh2^+/−^, Cdh3^−/−^, Cdh4^−/−^ and Cdh6^−/−^) failed to identify a compelling role for cadherins in the MET of NPCs *in vivo* (Mah et al., 2000). To characterize expression of cadherins, we performed *in vitro* bulk mRNA-sequencing of NPCs in low and high CHIR. *Cdh2* and *Cdh11* were expressed at high levels in low CHIR, and downregulated on induction in high CHIR (Fig. 4A). In contrast, *Cdh3*, *Cdh4* and *Cdh6* were upregulated in high CHIR conditions (Fig. 4A). Immunofluorescent staining of cultured NPCs confirmed the presence of Cdh2, Cdh4 and Cdh11 at the cell membrane before induction, and the appearance of Cdh3 and downregulation of Cdh11 at the membrane occurred in response to high CHIR-mediated induction of NPCs (Fig. 4B-E). In contrast, Cdh13 was not detected and Cdh6 was mostly confined to the GM130^+^ Golgi apparatus in induced NPCs, arguing against a role for Cdh6 in cell surface accessible adhesion complexes. (Fig. S4A-C). Immuno-analysis confirmed key features of predicted cadherin distribution in the NPC lineage in the developing kidney *in vivo* (tdTomato^+^ cells in Fig. 4F-I, Fig. S4D). A view of cadherin expression in the early human nephrogenic program showed a broadly similar expression for mouse and human cadherin genes (Fig. 4J; Fig. S4E).

**Fig. 5.**
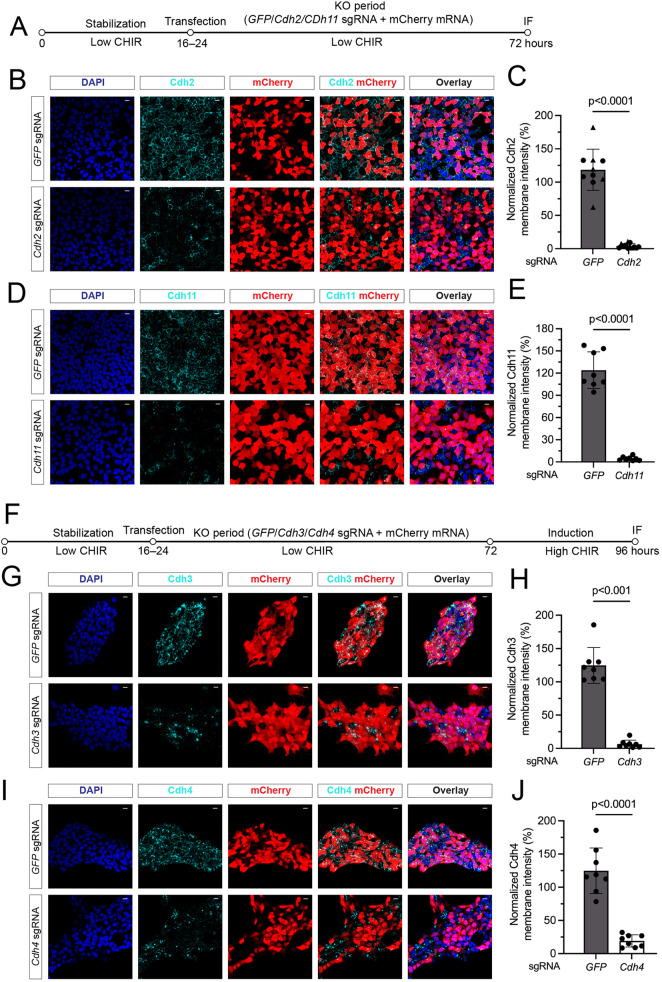
**Confirming the removal of individual cadherin proteins by the Cas9-sgRNA KO system.** (A) Schematic representation of the experimental protocol to remove Cdh2 and Cdh11 with a 48 h KO period. (B) Representative immunofluorescence images of Cdh2 removal (cyan) in mCherry transfected cells (red) with nuclear staining (DAPI, blue). Scale bars: 10 μm. (C) Quantifications of the membrane intensity of Cdh2 KO cells. Unpaired *t*-test. (D) Representative immunofluorescence images of Cdh11 removal (cyan) in mCherry transfected cells (red) with nuclear staining (DAPI, blue). Scale bars: 10 μm. (E) Quantification of the membrane intensity of Cdh11 KO cells (unpaired *t*-test). (F) Schematic representation of the experimental protocol to remove Cdh3 or Cdh4, including a 48 h KO period and a 24 h induction. (G) Representative immunofluorescence images of Cdh3 removal (cyan) in mCherry-transfected cells (red) with nuclear staining (DAPI, blue). Scale bars: 10 μm. (H) Quantification of the membrane intensity of Cdh3 KO cells (Mann–Whitney test). (I) Representative immunofluorescence images of Cdh4 removal (cyan) in mCherry transfected cells (red) with nuclear staining (DAPI, blue). Scale bars: 10 μm. (J) Quantification of the membrane intensity of Cdh4 KO cells (unpaired *t*-test). Data are mean±s.d.

**Fig. 6.**
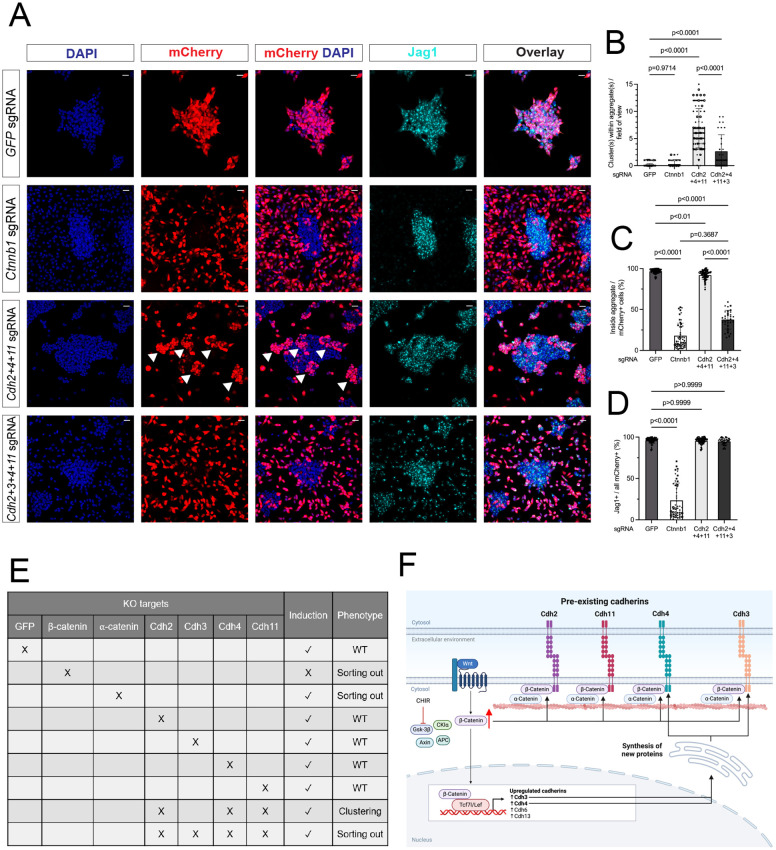
**Compound cadherin removal inhibits cell adhesion phenocopying α- and β-catenin removal; however, it maintains nephron progenitor cell transcriptional induction.** (A) Representative images of CTRL (GFP sgRNA), β-catenin-KO (positive CTRL) and pre-existing cadherin KO (Cdh2, Cdh4 and Cdh11), and QCKO (Cdh2, Cdh3, Cdh4 and Cdh11) showing cellular behavior (mCherry, DAPI) and induction (Jag1). Arrowheads highlight transfected nephron progenitor cell (NPC) clustering within aggregates. Scale bars: 25 μm. (B-D) Quantification of changes in cell adhesion [as the number of transfected cell cluster(s) within aggregates (B)], cell-sorting phenomenon [by percentage of cells within aggregates (C)] and induction rate of NPCs [by Jag1 expression in cadherin KO experiments (D)]. Data were tested for statistical significance using an ordinary one-way ANOVA (B) or a Kruskall-Wallis test (C,D) [three to six biological replicates (different symbol shapes); one or tow technical replicates (different colored of symbols)]. Data are mean±s.d. (E) Summary of the effects of single and compounded cadherin-catenin complex KOs on induction of NPCs and cellular behavior. (F) Proposed mechanism of the NPC aggregation regulated by Wnt/β-catenin via the cadherin-catenin complex. This models the initial step of nephrogenesis *in vivo*. Created with BioRender.com.

To test a potential role for the cadherins of interest, we identified effective sgRNA KOs for each cadherin, demonstrating complete removal of detectable Cdh2 and Cdh11 before high CHIR addition 48 h post-transduction, and 90-95% removal of Cdh3 and Cdh4 when assaying post-induction (Fig. 5A-J). Similar efficiencies were observed when combining gRNAs for multiple cadherin removals (Fig. S5A-C). Whereas targeting of individual cadherins did not alter the aggregation process or inductive process (Fig. S6A-E), combinatorial removal of Cdh2, Cdh4 and Cdh11 resulted in an interesting phenotype. Triple KO cells clustered at the boundary of cell aggregates in high CHIR, in contrast to control GFP gRNA KO cells or single cadherin KO cells, which dispersed randomly in the aggregate (Fig. 6A-C). Furthermore, membrane levels of Cdh3 were elevated on depletion of Cdh2, Cdh4 and Cdh11, consistent with a competitive stabilization of cadherins in the membrane (Fig. S6F,G). Strikingly, combining KO of Cdh3 with Cdh2, Cdh4 and Cdh11 (QCKO) prevented cell aggregation in high CHIR (Fig. 6A-C). Similar to α-catenin removal and distinct from β-catenin KO, QCKO cells were Jag1^+^, indicating a β-catenin-dependent transcriptional response in non-aggregated NPCs (Fig. 6A-D; these findings are summarized in Fig. 6E,F. Together, these results provide strong evidence for classic cadherin complexes in regulating the MET in the nephrogenic program.

### Analysis of modified forms of β-catenin in NPC aggregation

**Fig. 7.**
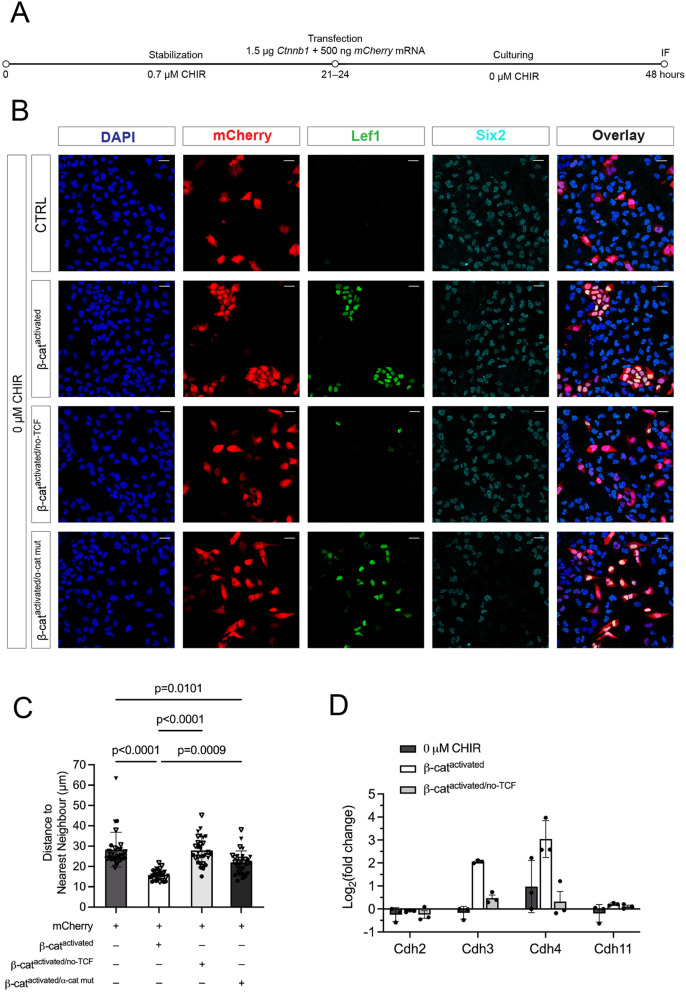
**Introduction of functionally altered β-catenin variants to nephron progenitor cells.** (A) Schematic of the experimental protocol to investigate the effects of introducing altered *Ctnnb1* mRNA to nephron progenitor cells (NPCs) and culturing them in no CHIR conditions. (B) Representative images of aggregation (mCherry, DAPI) and induction (Lef1 and Six2) of mCherry-transfected NPCs without *Ctnnb1* mRNA co-transfection (CTRL) or after the co-transfection of GSK3β-dependent degradation-resistant β-catenin (β-cat^activated^), β-catenin unable to bind Lef/Tcf factors (β-cat^activated/no-TCF^) or β-catenin mutated at the α-catenin-binding site (β-cat^activated/α-cat mut^). Scale bars: 20 μm. (C) Quantification of cell aggregation by plotting the distance to nearest neighbor of the transfected mCherry^+^ NPCs. Significance was tested using a Kruskall-Wallis test [two biological replicates (different symbol shapes), one or two technical replicates (different colored symbols)]. (D) RT-qPCR dataset showing selected Cdh genes after the co-transfection of *mCherry* mRNA with or without mutated *Ctnnb1* mRNA. Data are mean±s.d.

To investigate the direct role of β-catenin in cell adhesion and to avoid potential ‘off-target’ effects of GSK-mediated inhibition by CHIR, we used RNA-lipofection to introduce structurally altered mRNAs encoding mutant forms of β-catenin into NPCs (Table S2). In the absence of CHIR, introduction of *Ctnnb1* mRNA encoding a stabilized form of β-catenin that is resistant to GSK phosphorylation-directed proteosomal degradation (β-catenin^activated^, Table S2; Fig. 7A,B) resulted in a cell-autonomous induction, upregulating Lef1 and Jag1, and downregulating Six2, and the aggregation of induced NPCs (Bugacov et al., 2024; Fig. 7B,C; Fig. S7A,B). Introduction of point mutations disrupting β-catenin interactions with Lef1 and Tcf factors (β-catenin^activated/no-Tcf^, Table S2), and consequently the β-catenin-dependent transcriptional response (Fig. 7B; Fig. S7A,B), also prevent aggregation of NPCs (Fig. 7B). Aggregation could be quantified by measuring nearest-neighbor associations relative to mCherry mRNA transduced (negative control) and mCherry/β-catenin^activated^ mRNA co-transduced (positive control) samples (Fig. 7C). These data suggest transcriptional outputs are required for the β-catenin-dependent NPC aggregation process. Transcriptional activation of Cdh3 and Cdh4, early features of the Wnt-inductive program *in vivo* and *in vitro*, was dependent on the transcriptionally active form of β-catenin (Fig. 7D). To determine whether β-catenin interactions with α-catenin are crucial for cell aggregation, as predicted from experiments above, we generated point mutations within *Ctnnb1* to block β-catenin and α-catenin interactions (β-catenin^activated/α-cat mut^, Table S2). NPCs transduced with β-catenin^activated/α-cat mut^ failed to aggregate but nevertheless the dispersed NPCs underwent induction (Fig. 7B,C; Fig. S7A,B). Thus, aggregation, but not transcriptional induction of NPCs, is dependent on β-catenin interactions with α-catenin.

### Compound cadherin or α-catenin removal does not alter the transcriptional program within induced NPCs

**Fig. 8.**
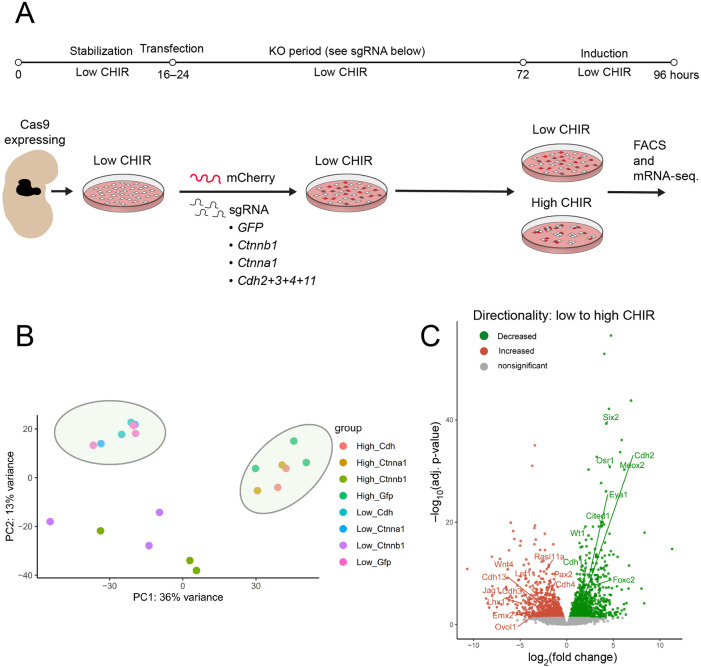
**Induction of nephron progenitor cells (NPCs) is independent of cadherin-mediated cell clustering in NPC culture.** (A) Schematic representation of workflow for transcriptional profiling of *Ctnna1* and *Ctnnb1*, and combined *Cdh2*, *Cdh3*, *Cdh4* and *Cdh11* knockout (QCKO) in NPC culture. (B) PCA plot of mRNA-seq transcriptional profiles of CTRL GFP KO (GFP sgRNA), β-catenin-KO (*Ctnnb1* sgRNA), α-catenin-KO (*Ctnna1* sgRNA) and QCKO in low and high CHIR conditions. PC1 (36%) variance relates to transcriptional induction status. (C) Volcano plot of low CHIR versus high CHIR DeSEQ2 bulk RNA-seq analysis examining control GFP gRNA samples in low and high CHIR (see Table S1). Highlighted genes include self-renewal-associated genes of interest downregulated in high CHIR conditions (green: *Six2*, *Cited1* and *Eya1*) and induction-associated genes of interest upregulated in high CHIR conditions (red: *Wnt4*, *Jag1*, *Lef1*, *Cdh3*, *Cdh4*, *Lhx1*, *Ovol1* and *Emx2*)*.*

To examine whether induction was independent of cell aggregation, we performed bulk mRNA-sequencing on two biological replicates of fluorescence activation-sorted genetically modified NPCs using the 48 h KO period followed by 24 h in high CHIR (Fig. 8A; Table S1). Principal component analysis comparing control (CTRL) samples targeting GFP with α-catenin KO, β-catenin KO and QCKO showed similar co-clustering amongst CTRL, α-catenin KO and QCKO experimental samples in each CHIR condition (Fig. 8B). In line with findings in the accompanying paper (Bugacov et al., 2024), CTRL KO samples targeting GFP showed an expected inductive response with the downregulation of NPC-associated genes and upregulation of large set of genes, including well recognized Wnt targets such as *Jag1*, *Lef1*, *Wnt4* and *Lhx1* (Guo et al., 2021; Fig. 8C; Table S1). Comparing induced (high CHIR) CTRL KO and α-catenin KO, only *Ctnna1* was identified as a differentially expressed gene (DEG; absolute log_2_FC cut off 0.5; *P*-adj cut off 0.5; Table S1). In CTRL KO versus QCKO, the only DEG was *Rasl11a* (Table S1). Thus, cell aggregation did not alter the β-catenin-dependent transcriptional response to high CHIR, which led to the conclusion that induction of the nephrogenic program is independent of cell reorganization, in the experimental model.

## DISCUSSION

Employing a culture system that reproduces key cellular behaviors and gene regulatory controls associated with Wnt-directed initiation of mammalian nephrogenesis, we provide multiple lines of evidence supporting a crucial role for cadherin/β-catenin/α-catenin adhesion complexes in the MET that underpins morphogenesis and patterning of the nephron (Fig. 6F). Cell-cell contacts between NPCs were Ca^2+^ sensitive and stabilized in high CHIR in a β-catenin process, requiring α-catenin and the partially redundant activities of four cadherins: Cdh2, Cdh3, Cdh4 and Cdh11. Examining cell-cell interactions in low and high CHIR suggests that uninduced and induced NPCs actively send out filopodia that make new cell contacts, which is consistent with *in vivo* observations (Combes et al., 2016; O'Brien et al., 2018). Existing cadherins likely mediate weak interactions in low CHIR, which are stabilized, leading to cell aggregation on induction in high CHIR. Cadherin switching has been observed in EMTs, including the emergence of migratory neural crest (Wheelock et al., 2008) and the progression of cholangiocarcinoma (Araki et al., 2011). The downregulation of Cdh11 has also been observed in the differentiation of osteocytes and adipocyte stem cells to more mature cell types (Alimperti and Andreadis, 2015).

Lefevre et al. (2017) catalogued cadherin expression within multiple bulk-mRNA sequencing data, highlighting *Cdh2*, *Cdh4* and *Cdh6* in the mesenchymal NPCs in the cap mesenchyme in the developing kidney, and *Cdh2*, *Cdh3*, *Cdh4*, *Cdh6*, *Cdh11* and *Cdh16* from renal vesicle to S-shape body stages. These findings are broadly consistent with data here. Furthermore, the single cell studies underscore the highly dynamic and regional interplay of different cadherins, consistent with continuing roles in the nephrogenic program beyond the initial induction and aggregation events that are the focus of this study. Zebrafish studies of spinal cord assembly highlight the important role played by cell type-specific combinatorial expression of different classes of cadherins, by creating a differential adhesion code in organizing the developing spinal cord in response to a sonic hedgehog morphogen gradient (Tsai et al., 2020). In addition to cadherin-directed cellular adhesion, the appearance of desmosomal proteins in early NPC clusters *in vivo* points to a potential role for desmosomes in adhesive cell interactions (Garrod and Fleming, 1990).

Despite some considerable efforts to identify developmental roles for cadherins in the kidney either individually or in compound mutant studies (Dahl et al., 2002), the only reported phenotypes from mutant studies is attenuated nephrogenesis in conjunction with abnormal ureteric branch organization in *Cdh4* mutants (Dahl et al., 2002). Here, disruption of the ureteric branches, the source of the primary Wnt9b signal that induces nephrogenesis, confounds a simple interpretation of Cdh4 action in the nephron-forming program (Carroll et al., 2005). Cdh6 mutants are reported to have later phenotypes with altered renal vesicle polarity and a loss of interconnection of nephrons to the ureteric epithelial network (Mah et al., 2000). Both *Cdh4* and *Cdh6* loss-of-function mutants are viable (Mah et al., 2000; Dahl et al., 2002). Antibody interference studies or kidney organ cultures with anti-Cdh6 antibodies have been reported to inhibit MET of NPCs (Cho et al., 1998). However, this finding is inconsistent with Cdh6 mutant analysis above. Furthermore, the absence of Cdh6 at the cell membrane at the aggregation stage *in vivo* (Fig. S4D) suggests that Cdh6 does not play an early role in NPC cell aggregation.

Interestingly, altering cadherin levels, by the KO of *Cdh2*, *Cdh4* and *Cdh11*, dramatically alters cell associations within cell aggregates, which suggests that differing levels or distinct forms of cadherins control adhesiveness, cell sorting and self-organization in tissue morphogenesis (Steinberg, 2007). A clustering phenotype has been described in other stem cell systems (Tse et al., 2021). Stem cell-directed human kidney organoids undergo a Wnt-induced MET and subsequent polarization of renal vesicles in response to a brief high CHIR stimulation (Glykofrydis et al., 2021; Morizane and Bonventre, 2017). Thus, the organoid model could be an additional informative system with which to address the question of how a Wnt pulse leads to complex cellular organization (Nishinakamura, 2023; Nishinakamura, 2019), and it could provide a powerful platform for studying cadherin-directed cell association, regional patterning and tissue organization. However, our study highlights the experimental challenge to mechanistic dissection of multiple overlapping cadherin domains.

Multiple lines of evidence demonstrate that disrupting cell aggregation did not alter the high CHIR and Wnt-mediated transcriptional component of the NPC inductive response. Although α-catenin forms a dynamic link between membrane-localized cadherin-catenin complexes and the actin cytoskeletal network (Shapiro and Weis, 2009; Yamada et al., 2005), a transcriptional role has been posited for α-catenin in *Xenopus* development (Sehgal et al., 1997) and in mammalian cell studies (Daugherty et al., 2014; Giannini et al., 2000). However, our findings that α-catenin-KO NPCs were transcriptionally almost identical to wild-type NPCs argues against a transcriptional role for α-catenin in NPC programs, in line with studies of α-catenin in the brain (Lien et al., 2006). Thus, although β-catenin links the MET to the inductive transcriptional response after Wnt-mediated activation of the nephrogenic program, these distinct cellular processes can be separated genetically and continue independently. A similar concentration of CHIR invoking cell aggregation in NPCs leads to Cdh2- and β-catenin-driven aggregation of mouse embryonic stem cells (Sineva and Pospelov, 2010).

Although cadherins undergo heterophilic or homophilic interactions (Takeichi, 2023), our genetic analysis suggests a high degree of functional redundancy among cadherins in the aggregation of induced NPCs. However, the situation *in vivo* may be different; this can only be resolved through compound mutant studies of the highlighted Cdh genes, specifically within NPCs in the developing kidney, which is a technical challenge. Genetic removal of α-catenin may provide an insight into the general role of cadherin complexes. The similar phenotypes resulting from multiple loss of cadherins, i.e. expression of an activated β-catenin mutant that is unable to associate with α-catenin, and α-catenin removal point to conventional cadherin complex assembly in the MET.

Interestingly, expression studies indicate that Cdh3 and Cdh4 are transcriptionally upregulated on induction by CHIR (Bugacov et al., 2024) and activated β-catenin (Fig. 7D), raising the question of whether the transcriptional program also contributes to NPC aggregation. Genetic removal shows that whereas knockdown of *Cdh2*, *Cdh4* and *Cdh11* results in NPC segregation to the outer edge of aggregates in high CHIR conditions, removal of *Cdh3* is required for complete dispersion of NPCs. *Cdh3* mRNA was present at low levels in low CHIR maintenance conditions and expression elevated markedly on high CHIR-mediated induction (Fig. 4A); indeed, Cdh3 is only detected post-induction (Fig. 4C). Together these results are consistent with a transcriptional role for *Cdh3* activation in the cell aggregation process. Further support for a transcriptional input to cell aggregation comes from the finding that the point mutations introduced into an activated form of β-catenin to abolish the β-catenin-mediated transcriptional response also removed NPC aggregating activity. However, we cannot exclude the possibility that point mutations attenuating β-catenin interactions with Lef/Tcf factors may also attenuate the interactions of β-catenin with other components of the adhesion complex. Crystal structure studies have implicated Lys 435 as a crucial amino-acid in β-catenin interactions with both Lef/Tcf factors and Cdh1, and both transcription and adhesion are blocked when the charge at this position is reversed: Lys435 to Glu435 (Graham et al., 2000; Huber and Weis, 2001; Koelman et al., 2022). Although we substituted a neutral Ala in this position and blocked the transcriptional response, the effect of an alanine substitution on adhesion is unclear. We also note that *Ctnnd2*, which encodes δ-catenin, a protein that bridges and links cadherin complexes, is also a transcriptional target of the Wnt/β-catenin pathway (Bugacov et al., 2024). Thus, the transcriptional program may reinforce cell adhesion and control subsequent morphogenesis of the epithelial nephron.

The dual role of β-catenin raises the question of whether each function is served by unique or common pools of β-catenin (van der Wal and van Amerongen, 2020). Interestingly, in transcriptional profiling following removal of all four cadherins, we do not observe any significant change in the transcriptional response, despite the expected freeing of β-catenin associated with membrane cadherin partners. These observations argue against a common pool model. In contrast, the full epithelial transition of aggregated NPCs requires the downregulation of β-catenin transcriptional activity in NPC culture and in *in vivo* nephrogenesis (Park et al., 2012), and non-canonical autocrine signaling by Wnt4, which is itself a direct target of Lef/Tcf/β-catenin transcriptional complexes (Guo et al., 2021; Park et al., 2012; Tanigawa et al., 2011). The block to epithelial formation could be explained by the transcriptional requirement that limits β-catenin availability for the formation of epithelial cadherin complexes.

In *Xenopus*, overexpression of cadherins translocates β-catenin to the cell membrane, which inhibits β-catenin transcriptional activity (Fagotto et al., 1996; Heasman et al., 1994). In *Drosophila* the overexpression of full-length E-cadherin (Cdh2) or its dominant-negative truncated form result in a similar phenotype to the *wingless* (*Drosophila* Wnt-1 homologue) deficient flies (Sanson et al., 1996). Furthermore, in human cell lines, overexpression of soluble Cdh1 and Cdh2 cytoplasmic domains also inhibits transcription of a Lef1 reporter of canonical Wnt transcriptional complexes (Sadot et al., 1998). In these experiments, supra-physiological approaches with abnormally high levels of given factors add an additional complication to mechanistic interpretation. The *in vitro* NPC model, which replicates an *in vivo* event, is well suited to a focused analysis of β-catenin dynamics in cell aggregation and transcription.

Our studies provide a direct and strong link between Wnt pathway activation and the MET of NPCs. Interestingly, many studies have highlighted Wnt pathway activation regulating EMTs in normal development and cancer (Arkell et al., 2013; Ji et al., 2019; Schepers and Clevers, 2012; Xu et al., 2020). A potential explanation for these opposing roles lies in the differential targets of β-catenin-directed transcriptional programs. In our studies, high CHIR results in *de novo* expression of Cdh3, which may enhance cell-cell adhesion in the epithelial transition. Importantly, EMT is commonly associated with transcriptional upregulation of the transcriptional repressors Snail/Sna1 and Slug/Sna2, which are direct targets of canonical Wnt transcriptional complexes in these paradigms (Heuberger and Birchmeier, 2010; Wu et al., 2012; Yook et al., 2006). Snail and Slug are master regulators of transcriptional programs promoting EMT, in part through transcriptional silencing of cadherins stabilizing epithelial organization (Bolós et al., 2003; Cano et al., 2000). Thus, the morphological outcomes of canonical Wnt signaling input, i.e. MET or EMT, likely reflect distinct cell type-specific epigenetic programming of Wnt-responsive cells.

In conclusion, our findings support a dual role for β-catenin in transcriptional programming and MET of NPCs. However, we note that the role of Wnt signaling and β-catenin is complex in the full transition of aggregated NPCs to an epithelial renal vesicle – the precursor for each nephron. Epithelial formation and progressive development of the nephrogenic program *in vivo* (Park et al., 2007) and *in vitro* (Park et al., 2012) requires the downregulation of β-catenin-dependent canonical Wnt signaling. Furthermore, there is evidence that Wnt4, a prominent target of the primary Wnt9b inductive response switches Wnt signaling in aggregated cells from canonical to non-canonical signaling that is associated with the epithelial transition (Tanigawa et al., 2011). These findings have significance beyond development to Wilm's tumor, a pediatric kidney cancer, in which a marked expansion on non-epithelial blast population sharing features with early induced NPCs associates with gain-of-function mutations in β-catenin (Spreafico et al., 2021).

## MATERIALS AND METHODS

### Animals

All animal-related research was reviewed and approved by the Institutional Animal Care and Use Committees (IACUC) at the University of Southern California, and experimental work was conducted accordingly. Midday on the morning of detection of a vaginal plug was considered to be the E0.5 timepoint. Mouse embryos were recovered for NPCs isolation or histological section analysis at E16.5 stage. Wild-type NPCs were isolated from SWR/J mice. KO experiments were performed with NPCs derived from crosses of Gt(ROSA)26Sor^tm1.1(CAG-cas9*,-EGFP)Fezh^/J (Platt et al., 2014) and SWR/J mice or matings set up with B6.129-*Ctnnb1^tm2Kem^*/KnwJ (Brault et al., 2001). Six2-TGC-tdTomato/Hoxb7-Venus kidneys were generated by crossing male Tg(Six2-EGFP/cre)1Amc/J (Kobayashi et al., 2008) mice with *Rosa26^tdTomato^* [B6.Cg-*Gt(ROSA)26Sor^tm14(CAG-tdTomato)Hze^*/J] females (Madisen et al., 2010). Progeny were crossed with homozygous tgHoxb7-Venus [Tg(Hoxb7-Venus*)17Cos/J] females (Chi et al., 2009). For generating Movie 3 (a KO experiment with high-resolution imaging of membrane fluorescent reporter), mice constitutively expressing the Cas9-eGFP strain (Platt et al., 2014; IMSR_JAX:026179) were crossed with mTmG fluorescent reporter mice [Muzumbar et al., 2007; *t(ROSA)26Sor^tm4(ACTB-tdTomato,-EGFP)Luo^*/J].

### NPC isolation and culture

NPEM formulation and NPC isolation protocols were based on protocols of Brown et al. (2015), with modifications by Bugacov et al. (2024). After completing NPC isolation, NPCs were plated in 24-well CELLSTAR cell culture plates (for bulk-RNA-seq and live imaging; VWR, 82050-892), in µ-Slide 8 Well (for Movie 1; Ibidi, 80827) or in treated 24-well µ-plates (for immunofluorescence staining; Ibidi, 82426). Plates were coated with Matrigel (Corning, 354277) as described by Bugacov et al. (2024).

Seeding densities were as follows: 16-24 h stabilization and induction for 24 h, 300,000 cells/well; 16-24 h stabilization, 24 h KO and induction for 24 h, 100,000-150,000 cells/well; 16-24 h stabilization and 48 h KO, 150,000 cells/well; 16-24 h stabilization, 48 h KO and induction for 24 h, 50,000-100,000 cells/well; 16-24 h stabilization and 24 h overexpression, 150,000 cells/well; timelapse in µ-Slide 8 well chip, 16-24 h stabilization and induction for 24 h, 100,000 cells/well; timelapse in 24-well plate, 16-24 h stabilization, 24 h KO and induction for 24 h, 150,000 cells/well.

For the extracellular Ca^2+^ removal experiments, the following types of cell culture media were used with supplemented low (1.25 μM), high (5 μM) or 0.7 μM CHIR concentrations: DMEM/F-12, HEPES (Thermo Fisher Scientific, 11330-032), DMEM, high glucose, no glutamine, no calcium (Thermo Fisher Scientific, 21068028).

Wnt3a was manufactured by R&D Systems (1324-WN-010/CF) and used at 200 ng/ml for 24 h induction. Bi-specific antibody (a gift from the Garcia laboratory; Janda et al., 2017) was added at the indicated concentration.

### *In vitro* mRNA synthesis and cell transfection

Cre and mCherry mRNA synthesis and NPC lipofection followed the protocol described in detail by Bugacov et al. (2024). In this set of experiments, we added 500 ng of total mRNA (per transcript type) to one well of a 24-well plate in conjunction with sgRNA(s) in the KO experiments. For Movie 3, NPCs were transfected with Cre mRNA. β-Catenin mRNA was created using pcDNA6-N-3XFLAG-Ctnnb1 plasmid (Addgene, 123586). For T120 Y142 mutagenesis, custom gene synthesis was ordered from Genewiz. The DNA template for mRNA synthesis included linearization of plasmid DNA downstream of the stop codon with restriction endonuclease XhoI (New England Biolab, R0146L). Per 24 wells, 1.5 μg of β-catenin mRNA were added along with 500 ng of mCherry mRNA. Different β-catenin point mutations are listed in Table S2.

### CRISPR mediated gene removal

For KO experiment protocols, see Bugacov et al. (2024). sgRNAs used for 48 h KO period experiments were ordered from Synthego designed by CRISPR Design Tool (except GFP and *Ctnnb1*). The top four guides were triaged based on immunofluorescent staining KO efficiency. Synthesized gRNAs were reconstituted at 100 pmol/μl in TE buffer and stored at −20°C. Total sgRNA concentration for the KO experiments was 7.5 pmol/well for a 24-well plate. For the QCKO experiments, 1.875 pmol of each gRNA was added to each well and 2.5 pmol of each gRNA for triple KO cadherin experiments (*Cdh2*, *Cdh4* and *Cdh11*). mCherry mRNA was co-transfected at a concentration of 500 ng/well. All sgRNA sequences are listed in Table S2.

### FACS sorting, mRNA isolation and RT-qPCR

For detailed protocols, see Bugacov et al. (2024). Briefly, NPCs were washed with PBS and trypsinized at 37°C. After terminating the reaction, NPCs were resuspended in AutoMACS buffer with DAPI and DRAQ5 dyes. We sorted NPCs on BD FACS Aria IIu (BD Biosciences). RNA was isolated with RNeasy Micro Kit (74004, Qiagen). We used SuperScript IV VILO Master Mix (11766050, ThermoFisher) for reverse transcription. Luna Universal qPCR Master Mix Protocol (M3003, New England Biolab) was used for qPCR on ViiA 7 Real-Time PCR System with 96-Well Block (4453534, ThermoFisher). Primer sequences are listed in Table S2.

### Bulk RNA-sequencing

Library preparation and methods for data analysis followed the procedures of Bugacov et al. (2024). To identify differentially expressed gene lists, normalized count tables were inputted to DeSEQ2 (Love et al., 2014) using a Log_2_FC cut off +1 or −1, and an adjusted *P*-value no greater than 0.05. ggplot and complex heatmap functions in R (Gu et al., 2016) were used to visualize data and the Benjamini-Hochberg correction (False Discovery Rate) was implemented with gene normalized counts greater than or equal to 10 using a hypergeometric test.

### Immunofluorescent analysis

Immunofluorescent analysis of proteins followed the procedure outlined by Bugacov et al. (2024) with modifications. NPCs were cultured on tissue culture-treated 24-well µ-plates (Ibidi, 82426), washed with pre-warmed PBS and fixed with ice-cold 4% PFA in PBS on ice for 30 min, then washed with PBS twice. For time series experiments, fixation took place at 0, 6, 12 and 24 h timepoints. For nuclear labeling, cells were incubated with 1:10,000 Hoechst 33342 (Thermo Fisher Scientific, H3570) in PBS for 10 min then in PBS alone. 24-well plates were kept away from light at 4°C before image acquisition. Primary and secondary antibodies are listed in Table S2.

### Image acquisition and quantification

Phase-contrast images were acquired by DLPlan Fluor 10×/NA 0.3 dry, Ph1 DL objective connected toa Mono Camera Nikon DS-Fi3 in Nikon ECLIPSE Ts2R inverted research microscope (Nikon Instruments). Confocal images were collected using Leica SP8-X confocal fluorescence imaging system (Leica Microsystems) with 40×/1.3 NA oil, HC PL APO CS2 or 63×/NA 1.4 oil, HC PL APO CS2 objectives in 1024×1024 pixels. Bright-field images were acquired using a Leica Thunder wide-field microscope with a 40×/NA 0.60 dry, HC PL FLUOTAR L objective. When area of interest did not fit into one scanning plane, *z*-stacks were acquired and represented as stacked images.

Timelapse recording for Movie 1 was recorded with a 40×/1.3 NA oil, HC PL APO CS2 on Leica SP8-X. Movies 2 and 3 were respectively acquired with a 10×/NA 0.45 dry, HC PL APO and a 40×/NA 0.60 dry, HC PL FLUOTAR L on a Leica Thunder widefield microscope. During timelapse image acquisition, homeostatic conditions were maintained with Ibidi stage top incubation system (Ibidi, 10720).

Images were quantified (‘surface’, ‘cell’ and ‘spots’ modules) in Imaris microscopic image analysis software (version 10.0, Oxford Instruments). Procedures applied to quantifying the removal of membrane α-catenin, Cdh2, Cdh3, Cdh4 and Cdh11 proteins have been described previously (Bugacov et al., 2024). Fluorescent intensity histograms were created for ‘surface’, ‘cell’ and ‘spots’ modules for mCherry channel to classify mCherry^+^ and mCherry^−^ NPCs based on steep intensity drop offs in the histogram. The criteria for assessing Jag1^+^ cells have been described previously (Bugacov et al., 2024). ‘Spots’ module was used for fluorescent intensity thresholding based on intensity histogram for Lef1 or Six2 channel to classify Lef1^+^/Lef1^−^ or Six2^+^/Six2^−^ cells in β-catenin transfection experiments, respectively.

‘Spots’ module was used to track the NPCs shown in Movie 1 to determine the number of mCherry^+^ cells outside the aggregates, and to measure the distance to the nearest neighbor (cells were excluded from evaluation if their nearest neighbor were more distant than mean+2s.d. at Fig. 1F). Cell tracks in Movie 1, cell-cell contact numbers and durations were manually annotated, as well as membrane and filopodia contact events (see examples in Fig. S1E). Cellular height, cellular volume, cellular size, nuclear height, nuclear volume were quantified by ‘surface’ or ‘cell’ modules.

Cells were considered to be sorted from cell aggregates if the cell body showed no membrane or filopodial contact with the Jag1^+^ NPC aggregate. Clusters within the aggregates of Cdh2+Cdh4+Cdh11 KO experiments were defined based on the following criteria: (1) at least five transfected cells were adjacent to each other; (2) a maximum of one non-transfected cell within the transfected cell cluster; (3) the cluster was entirely surrounded by non-transfected cells and/or the cluster was positioned at the boundary of an aggregate. Clusters were manually annotated and counted with the ‘spots’ module.

We applied median filter and background subtraction in Imaris for Movies 1-3, and processed by computational clearing, median filtering and thresholding in LASX. The frames of Movie 3 have been stabilized in ImageJ.

### Statistics and data plotting

We evaluated the normal distribution of datasets by D'Agostino-Pearson test. When we compared two independent groups with a normal distribution, significance levels were determined using an unpaired *t*-test. With a non-normal distribution, we used a Mann–Whitney test. Comparing more than two groups with a normal distribution, we performed a one-way ANOVA. With multiple samples and a non-normal distribution, *P*-values were calculated with a Kruskall-Wallis test. Mixed effect statistical analysis was applied for Fig. S1D and Fig. 2H datasets. Data are plotted as mean±s.d. unless otherwise specified in figure legends. Statistical tests were considered significant at *P*≤0.05. Graphs were plotted using Prism 9.4 (GraphPad by Dotmatics). The summary schematic was created with BioRender.com.

## Supplementary Material

10.1242/develop.202303_sup1 Supplementary information

Table S1. Summary table of bulk RNA-sequencing data corresponding to Fig. 8.

Table S2. Details of sgRNA manufacturers and sequences, and primary and secondary antibodies, mutated β-catenin forms.
