# Supplementary material for: Cadherin adhesion complexes direct cell aggregation in the epithelial transition of Wnt-induced nephron progenitor cells
Source: Development. 2024 Sep 30;151(18):dev202303. doi: 10.1242/dev.202303 (PMC11463967; doi:10.1242/dev.202303)
Supplement: Supplementary information [file develop-151-202303-s1.pdf]

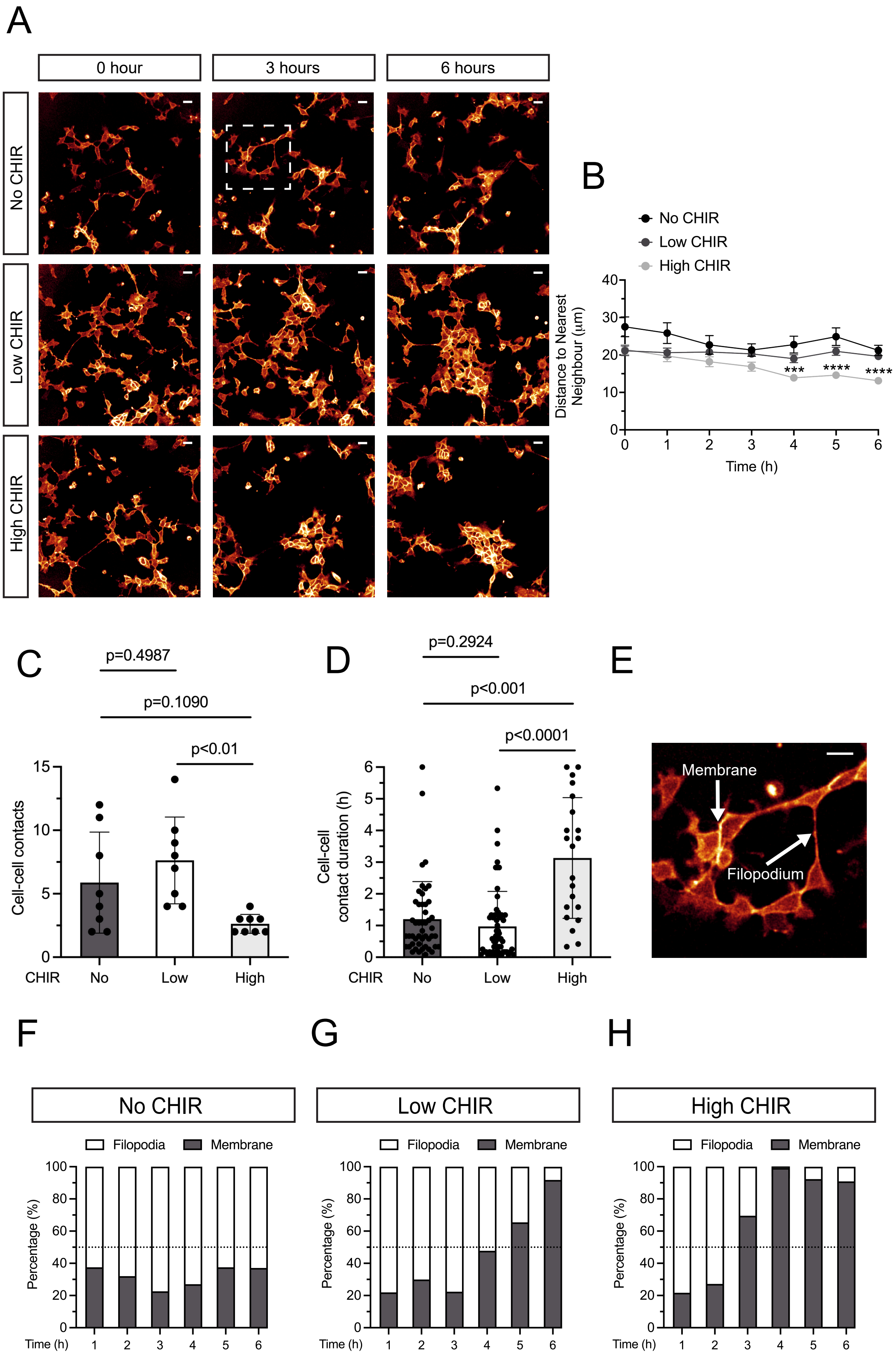

Development • Supplementary information

Fig. S1. ***Detailed analysis of individual NPC behavior with increased Wnt stimulus***

(A) Time lapse stills of NPCs with membrane tdTomato fluorescent reporter cultured in no (0 μM) CHIR, low (1.25 μM) CHIR and high (5 μM) CHIR over 6 h. Images are derived from Vid.S1. Squared area is magnified in Fig. S1E. Scalebars are 20 μm.

(B) Quantification of distance to nearest neighbor cell over the culture period above. Statistical analysis: mixed effect analysis. Graph is plotted as mean ± SEM. For each field of view or condition cell position was annotated manually. Average evaluated number of cells/field of view: no CHIR=49, low CHIR=93, high CHIR=84).

(C–D) Quantification of contact numbers per cell and contact duration in NPC cultures. Datapoints represent eight individual cells at starting timepoint field of view/condition (C), the intercellular contact durations of these eight cells are displayed in (D) Statistical tests were ordinary one-way ANOVA and Kruskall-Wallis test, respectively.

(E) Representative images of the types of cell-cell contacts: filopodium or membrane-membrane contacts.

(F–H) Quantification of filopodia and membrane-membrane contact ratio over 6 h in no CHIR, low CHIR and high CHIR conditions. The dotted grid line labels 50%.

Development • Supplementary information

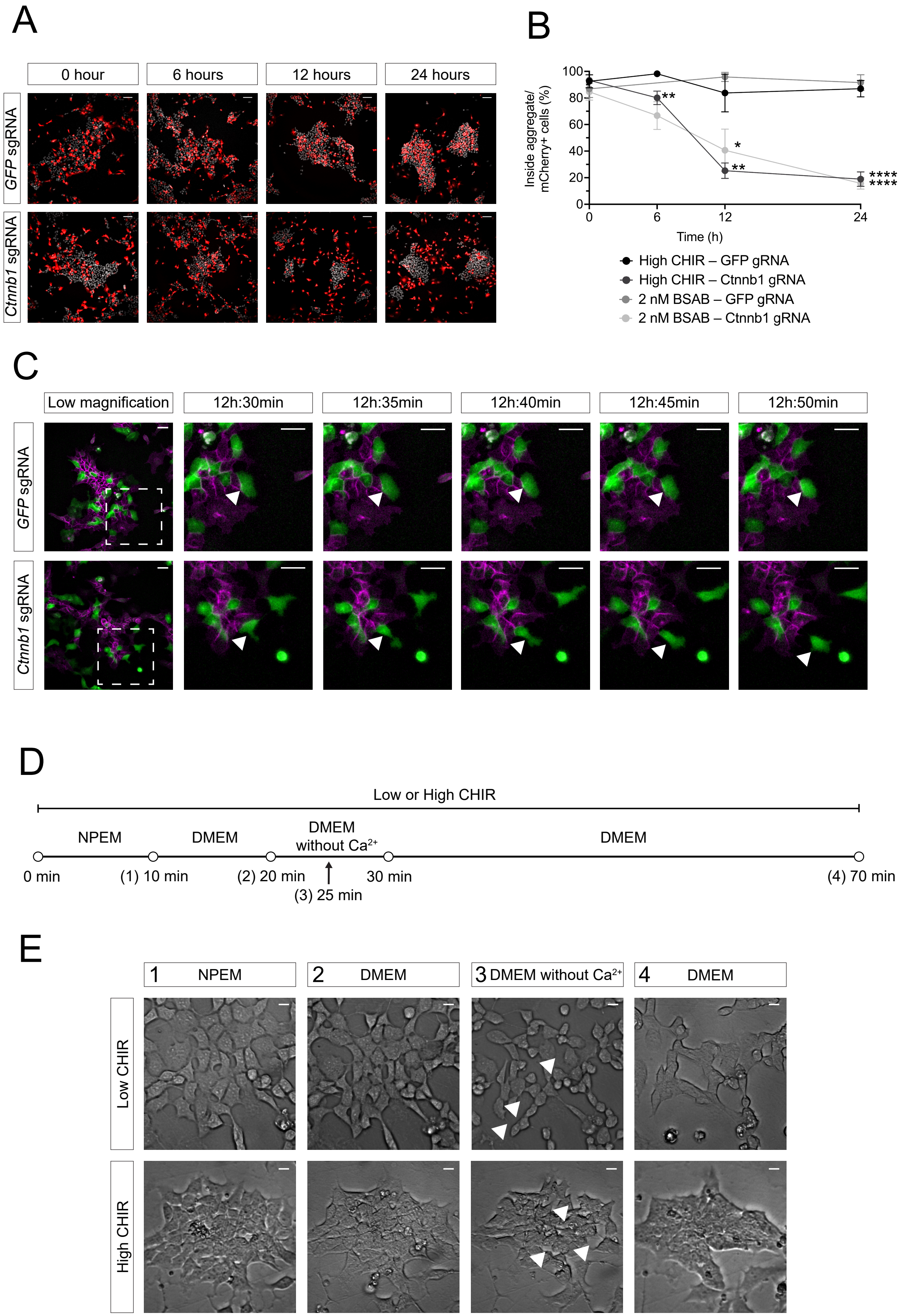

Development • Supplementary information

Fig. S2. ***Detailed analysis of cell sorting following ß-catenin KO and Response of NPCs to Ca^{2+} removal***

(A) Representative images of time series experiment of CTRL (GFP sgRNA) and β-catenin-KO (Ctnnb1 sgRNA) conditions when NPCs were fixed and IF stained at 0, 6, 12 and 24 h after the initiation of induction. Scale bars are 50 µm.

(B) Quantification of time series experiment by % of cells within aggregates. n=5–10 field of views/ well, 1–2 biological replicates, 1–2 technical replicates, mixed effect statistical analysis.

(C) Still images showing individual cell behaviour during aggregation and sorting from timelapse Vid. S3. NPCs isolated from mTmG x Cas9-eGFP mice are co-transfected with Cre mRNA and sgRNA targeting GFP (CTRL) and β-catenin (Ctnnb1). Scale bars are 10 µm.

(D) Schematic representation of the experimental protocol to examine the role of extracellular Ca^{2+} in NPC culture.

(E) Representative images of E16.5 NPCs cultured in NPEM (CTRL), DMEM (negative CTRL), DMEM without Ca^{2+} (experimental) and the re-addition of DMEM in low and high CHIR conditions. The loss of cell-cell contacts are labelled with white arrowheads. Scale bars are 10 µm.

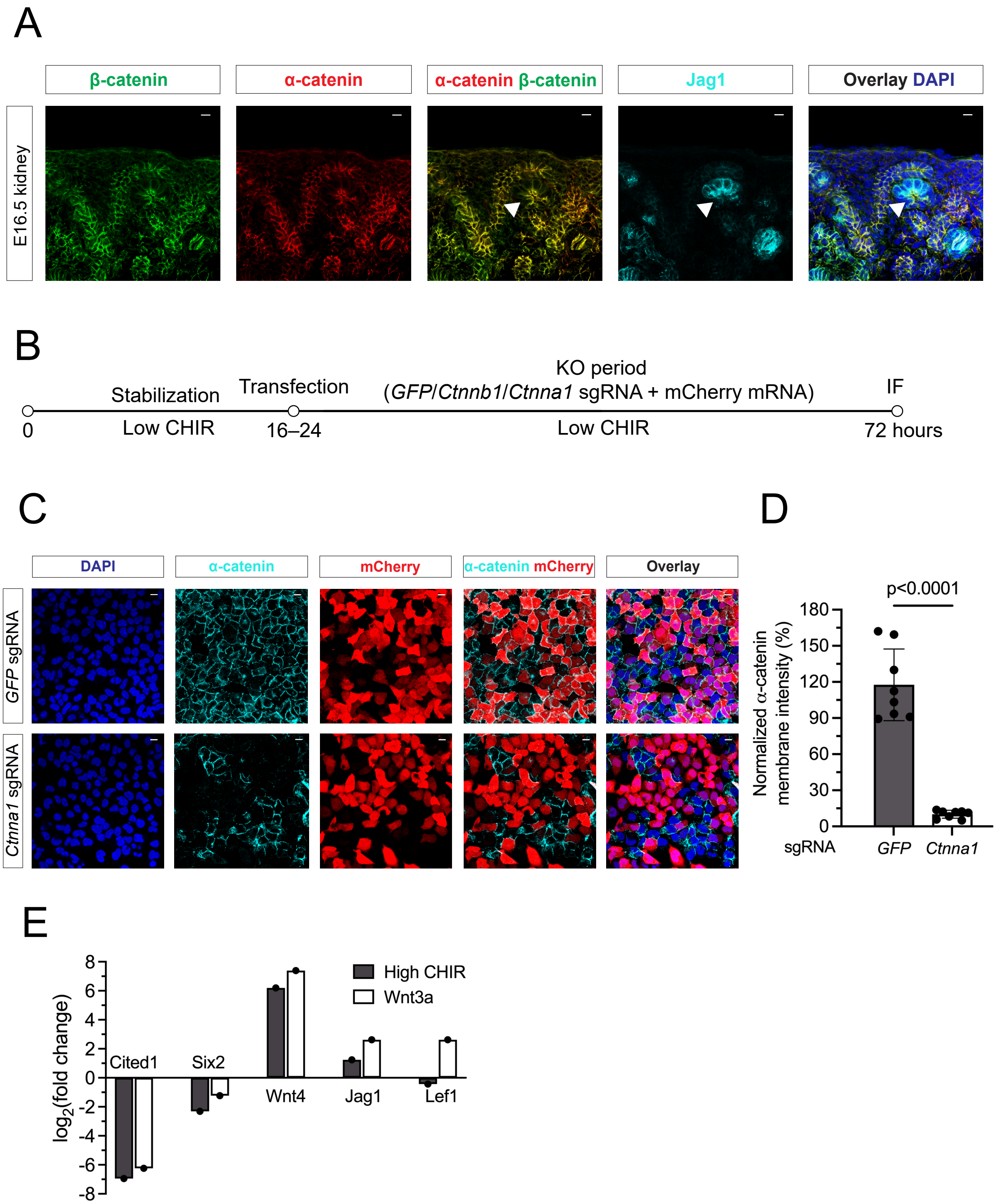

Development • Supplementary information

Fig. S3. ***In vivo characterization of α-catenin and in vitro validation of sgRNA-Cas9 system for CTRL and α-catenin-KO condition***

(A) Representative images of IF co-staining of α- and β-catenin (α-catenin: green, β-catenin: red) and the induction marker Jag1 (cyan) in wild-type E16.5 kidney. Arrows mark RV with apical accumulation of catenins and strong distal expression of Jag1. Scale bars are 10 µm.

(B) Schematic representation of the experimental protocol to validate removal of α-catenin.

(C) Representative IF images of α-catenin removal (cyan) in mCherry transfected cells (red) with nuclear staining DAPI (blue). Scale bars are 10 µm.

(D) Quantifications of the membrane intensity of α-catenin KO cells. Unpaired *t* test.

(E) RT-qPCR dataset showing comparable upregulation of induction genes and the downregulation of self-renewal gene network after 24 h of incubation with high CHIR and Wnt3a (200 ng/ml).

Development • Supplementary information

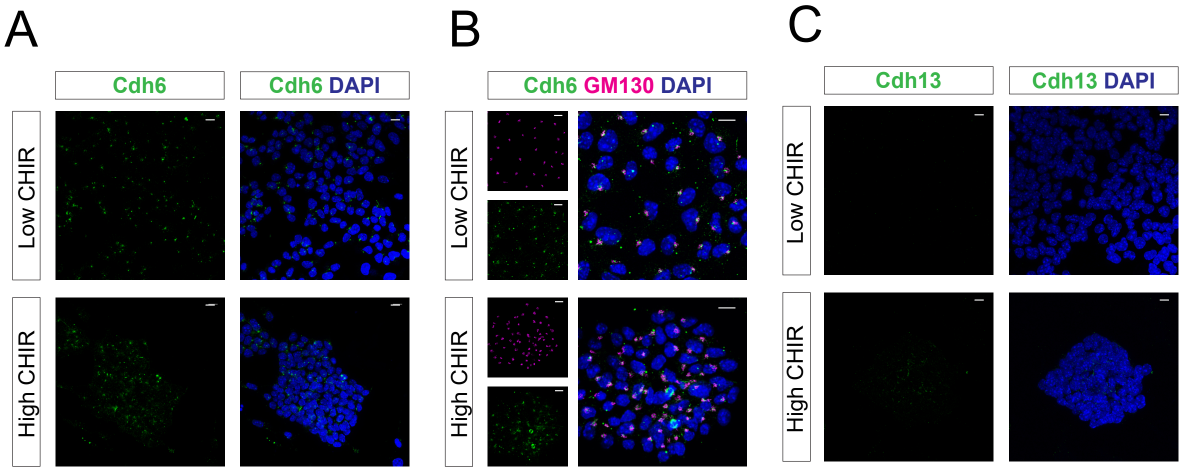

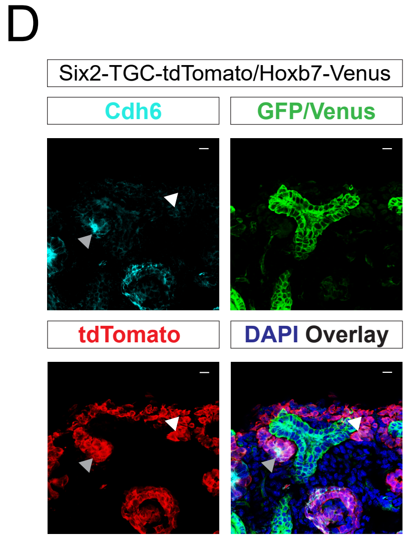

E

Gene	Name	*In vitro*				*In vivo*				Dahl *et al.*, Mol Cell Biol., 2002
		Uninduced NPC (Low CHIR)		Induced NPC (High CHIR)		Uninduced NPC		Induced NPC (PTA, RV)		
		Bulk RNA-seq.	IF	Bulk RNA-seq.	IF	sc. RNA-seq.	IF	sc. RNA-seq.	IF	
Cdh2	N-cadherin	+++	✓	++	✓	++	✓	++	✓	$Cdh2^{+/-}$
Cdh3	P-cadherin	–	–	++	✓	–	–	+	✓*	$Cdh3^{-/-}$
Cdh4	R-cadherin	+	✓	+++	✓	+	✓	++	✓	$Cdh4^{-/-}$
Cdh6	K-cadherin	–	✓**	++	✓**	–	–	+++	✓	$Cdh6^{-/-}$
Cdh11	OB-cadherin	+++	✓	++	✓	+++	✓	++	–	–
Cdh13	T-cadherin	–	–	+	–	–	N/A	+	N/A	–

Fig. S4. ***In vitro and in vivo analysis of mRNA and protein levels for additional cadherins***

(A) IF staining of Cdh6 (green) in isolated E16.5 NPCs shows a punctate pattern of protein localization in low and high CHIR conditions.

(B) Co-labeling of Cdh6 and GM130 Golgi-marker (Cdh6: green, GM130: magenta) in NPCs from E16.5 kidneys cultured in low and high CHIR conditions. Scale bars are 10 μm.

Development • Supplementary information

(C) IF staining of Cdh13 (green) in isolated E16.5 NPCs shows weak, sporadic membrane localization restricted to high CHIR conditions.

(D) Representative images of IF staining of E16.5 Six2-TGC-tdTomato/Hoxb7-Venus mouse kidneys highlighting indicated cadherins (cyan), tdTomato (nephron lineage, red) and GFP: nuclear GFP highlights Six2 in NPCs while membrane GFP labels Venus reporter in the ureteric lineage. Cdh6 is not detected in uninduced NPCs but Cdh6 is present in the late RV (white and gray arrowheads).

(E) Table summarizing *in vitro* and *in vivo* cadherin expression and protein levels in E16.5 mouse NPC culture and the E16.5 mouse kidney. (–) no expression/presence of mRNA/protein. + to +++: low to high expression/levels of mRNA or protein. ✓*: protein is present in distal RV. ✓**: protein is presented in Golgi-apparatus. RV: renal vesicle, PTA: pre-tubular aggregate

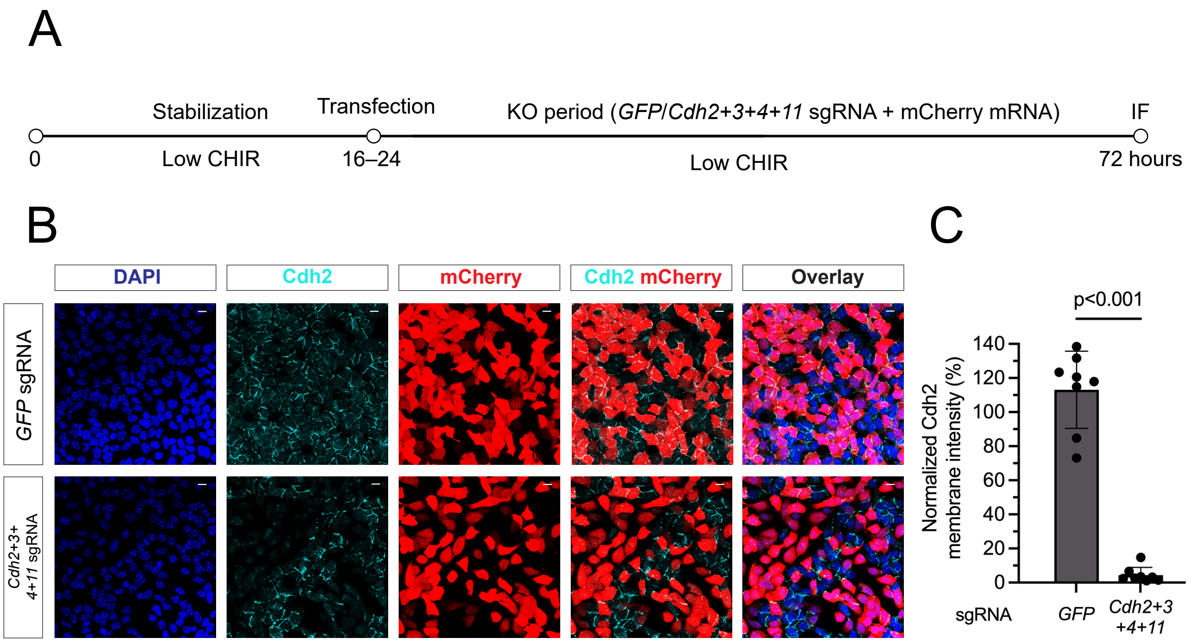

Fig. S5. ***Confirming the removal of Cdh2 protein removal in QCKO by the Cas9-sgRNA system***

(A) Schematic representation of the experimental protocol to remove Cdh2+3+4+11 including 48 h KO period.

(B) Representative IF images of Cdh2 removal (cyan) in mCherry transfected cells (red) with nuclear staining DAPI (blue). Scale bars are 10 μm.

(C) Quantifications of the membrane intensity of Cdh2 in QCKO cells. Mann-Whitney test.

Development • Supplementary information

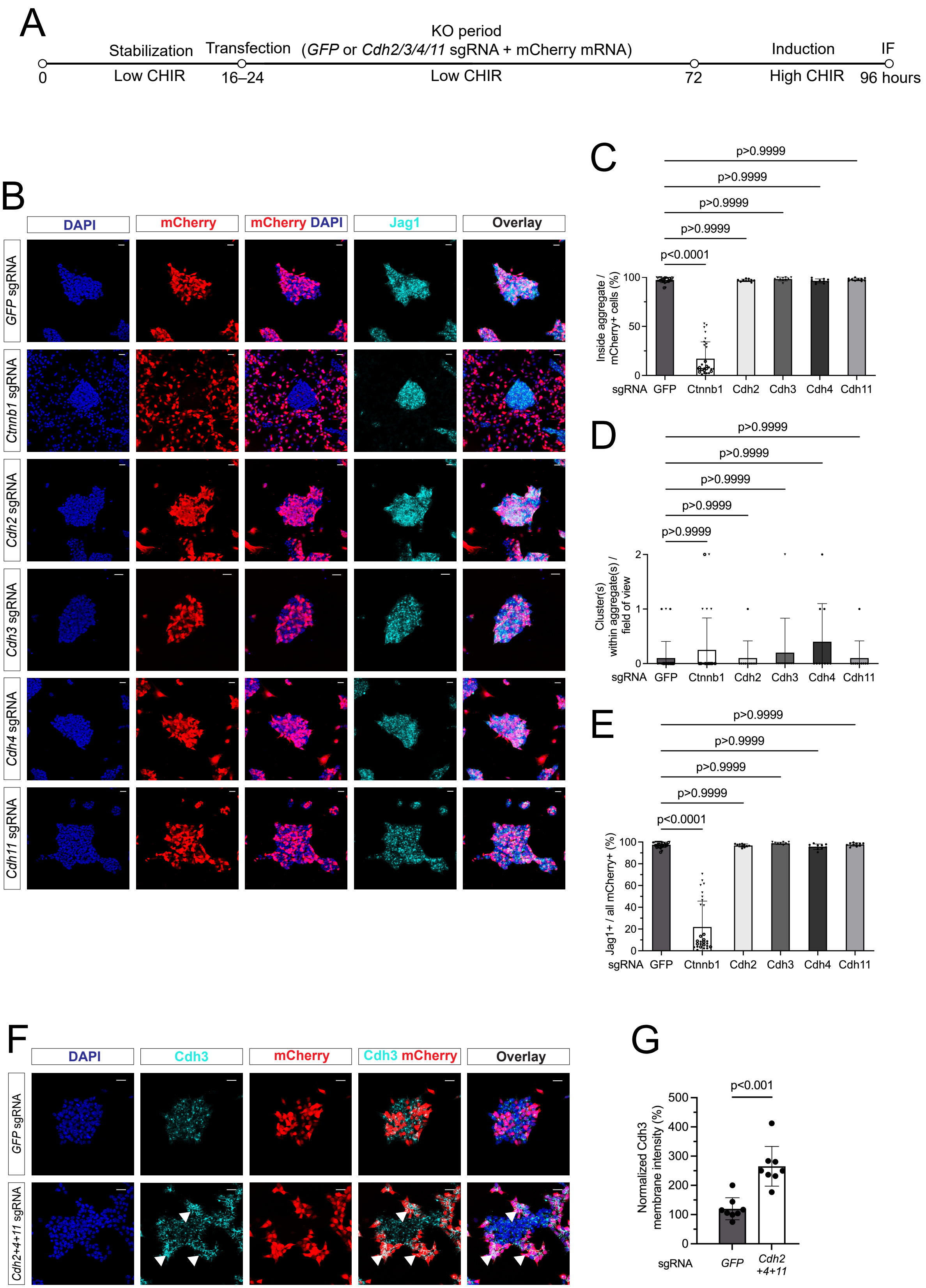

A
KO period
Stabilization
Transfection
(GFP or Cdh2/3/4/11 sgRNA + mCherry mRNA)
Induction
IF
0
Low CHIR
16–24
Low CHIR
72
High CHIR
96 hours
B
DAPI
mCherry
mCherry DAPI
Jag1
Overlay
GFP sgRNA
Ctnnb1 sgRNA
Cdh2 sgRNA
Cdh3 sgRNA
Cdh4 sgRNA
Cdh11 sgRNA
C
p>0.9999
p<0.0001
Inside aggregate / mCherry+ cells (%)
100
50
0
sgRNA
GFP
Ctnnb1
Cdh2
Cdh3
Cdh4
Cdh11
D
p>0.9999
Cluster(s) within aggregate(s) / field of view
2
1
0
E
p>0.9999
p<0.0001
Jag1+ / all mCherry+ (%)
100
80
60
40
20
0
F
DAPI
Cdh3
mCherry
Cdh3 mCherry
Overlay
GFP sgRNA
Cdh2+4+11 sgRNA
G
p<0.001
Normalized Cdh3 membrane intensity (%)
500
400
300
200
100
0
sgRNA
GFP
Cdh2 +4+11

Fig. S6. ***Individual cadherin removal does not influence high CHIR-dependent cell aggregation or induction of transcriptional targets***

(A) Schematic of the experimental protocol to investigate the effects of individual removal of Cdh2, Cdh3, Cdh4 or Cdh11 from E16.5 NPC cultures in high CHIR conditions.

(B) Representative images of negative CTRL GFP sgRNA, positive CTRL β-catenin-KO and individual cadherin KO in E16.5 NPC culture in high CHIR: DAPI, nuclei; mCherry^{+}, transduced NPCs, Jag1^{+}, induced NPCs. Scale bars are 25 μm.

(C–D) Quantification of changes in cell clustering of transduced NPCs. (C) percentage of transfected cells within aggregates. (D) number of within cell aggregate clusters of transfected NPCs. Biological replicates are represented by different symbol shapes and technical replicates are highlighted by different fill colors. Statistical analysis using a Kruskall-Wallis test.

(E) Percentage of Jag1^{+} transfected NPCs in cadherin KOs. Biological replicates are represented by different symbol shapes and technical replicates are highlighted by different fill colors. Statistical analysis using a Kruskall-Wallis test.

(F) Representative images of Cdh3 IF staining GFP sgRNA and β-catenin-KO showing elevated membrane intensity of Cdh3 (cyan) on Cdh2, Cdh4 and Cdh11. Scale bars are 25 μm.

(G) Quantification of the membrane intensity of Cdh3 in CTRL and Cdh2, Cdh4 and Cdh11 KO cells. Mann-Whitney test.

Development • Supplementary information

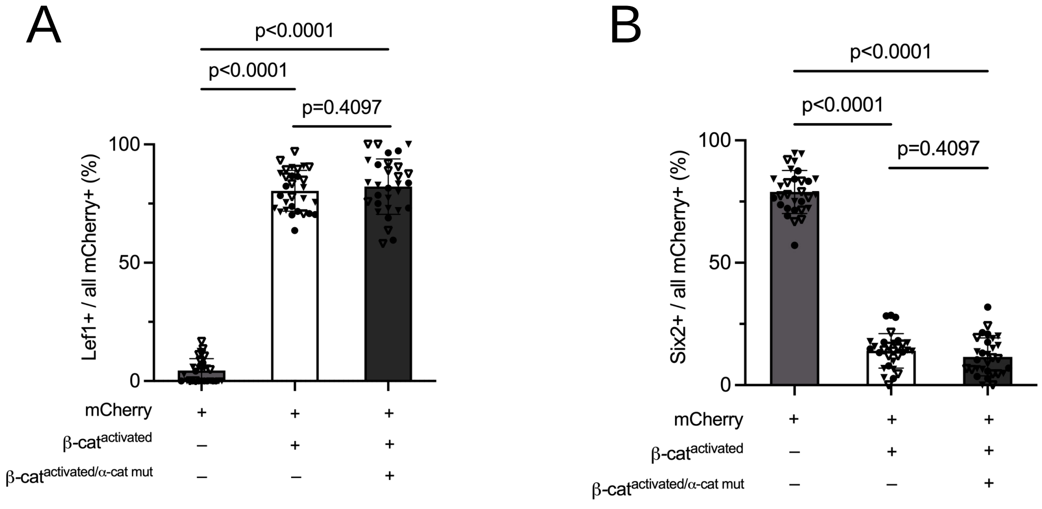

Fig. S7. ***Quantification of induction after introducing functionally altered β-catenin variants to NPCs***

(A) Quantification of induction by Lef1 expression percentage of NPCs after the transfection of mutated β-catenin forms corresponding to Fig. 7B. Ordinary one-way ANOVA. 2 biological replicates (different symbol shapes), 1–2 technical replicates (different fill colors of symbols).

(B) Quantification of self-renewal associated Six2 expression percentage of NPCs after the transfection of mutated β-catenin forms corresponding to Fig. 7B. Ordinary one-way ANOVA. 2 biological replicates (different symbol shapes), 1–2 technical replicates (different fill colors of symbols).

Table S1. Summary table of bulk RNA-sequencing data corresponding to Fig. 8.

Available for download at
https://journals.biologists.com/dev/article-lookup/doi/10.1242/dev.202303#supplementary-data

Table S2. Details of sgRNA manufacturers and sequences, and primary and secondary antibodies, mutated β-catenin forms.

Available for download at
https://journals.biologists.com/dev/article-lookup/doi/10.1242/dev.202303#supplementary-data

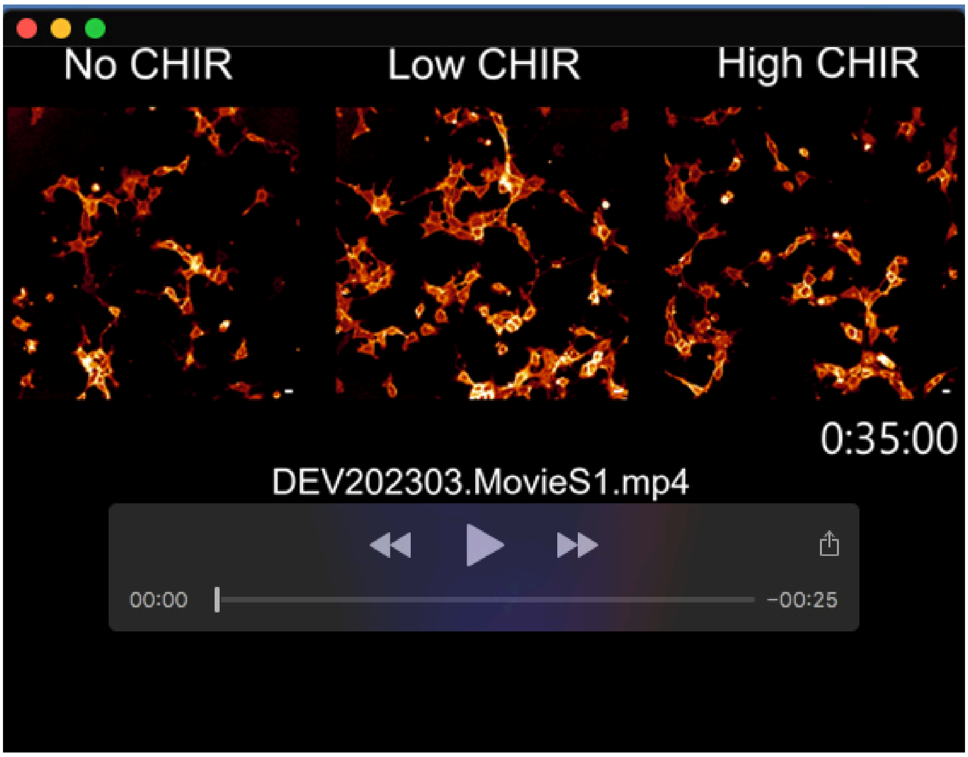

Movie 1. Timelapse recordings showing individual NPC behavior in no (0 µM), low (1.25 µM) and high (5 µM CHIR) conditions: Fig. S1 shows snapshots from this video data. NPCs are isolated from mT/mG mice and show membrane tdTomato reporter activity. Scale bars are 10 µm.

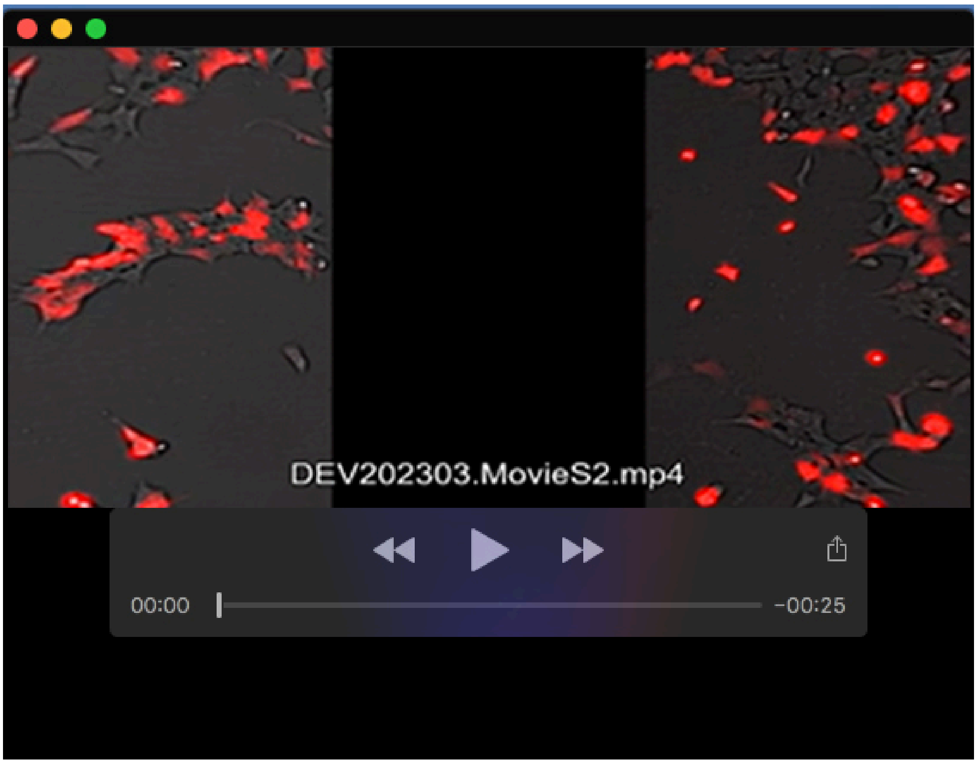

Movie 2. Timelapse recordings showing NPC cell sorting comparing mCherry transfected NPCs (red) receiving either GFP sgRNA (CTRL) or *Ctnnb1* sgRNA (β-catenin-KO). mCherry signal is projected on the brightfield channel. Recording covers 24 h period following addition of high CHIR. Scalebars are 100 µm.

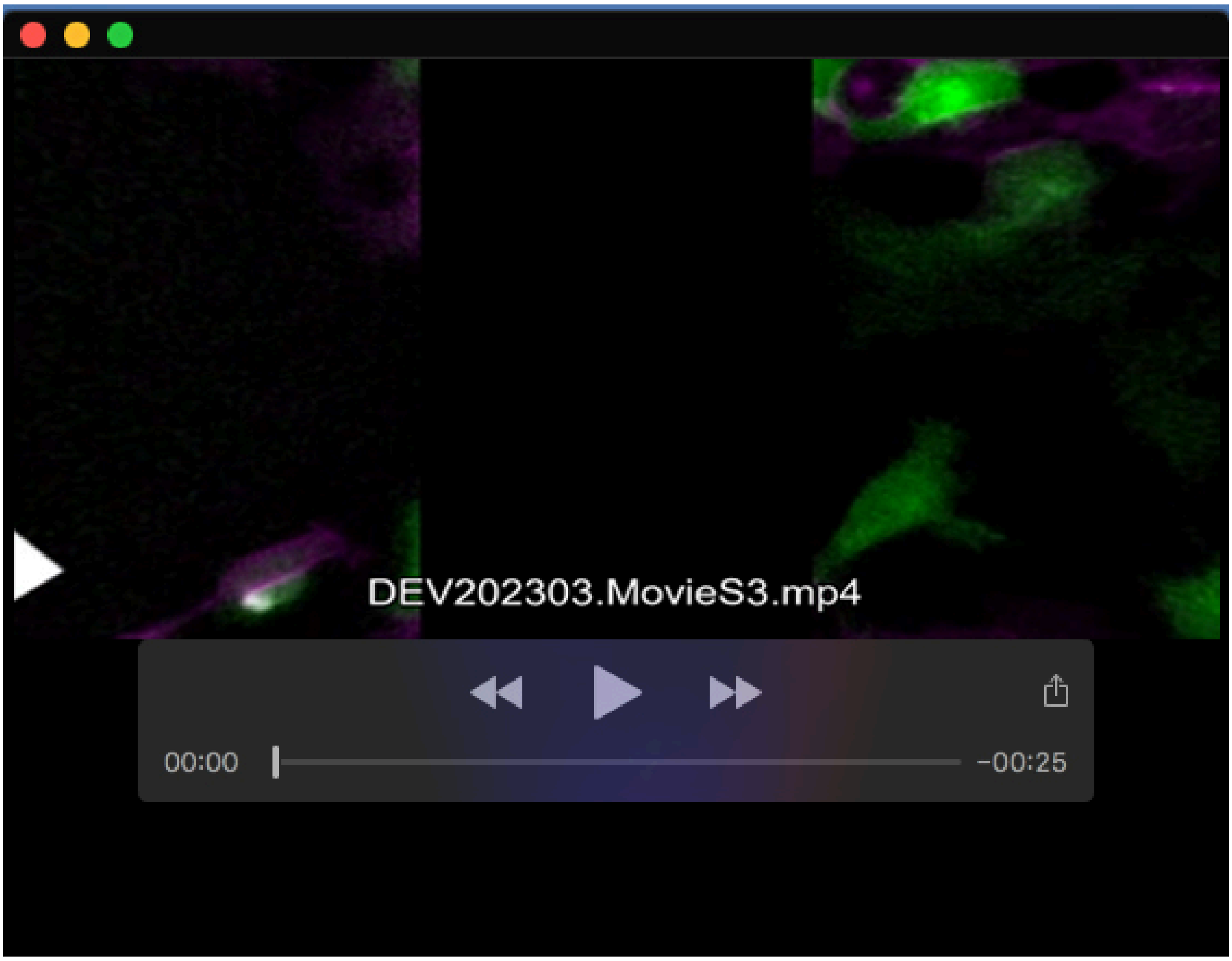

Movie 3. Timelapse recordings showing high-resolution views of cell sorting by β-catenin-KO cells in high CHIR (views related to Figure S2C). Non-transfected cells are magenta and transfected NPCs are green. Arrows highlight representative cellular events. Scalebars are 10 µm.

Development • Supplementary information
